# Gene Expression Profile of Neuronal Progenitor Cells Derived from hESCs: Activation of Chromosome 11p15.5 and Comparison to Human Dopaminergic Neurons

**DOI:** 10.1371/journal.pone.0001422

**Published:** 2008-01-09

**Authors:** William J. Freed, Jia Chen, Cristina M. Bäckman, Catherine M. Schwartz, Tandis Vazin, Jingli Cai, Charles E. Spivak, Carl R. Lupica, Mahendra S. Rao, Xianmin Zeng

**Affiliations:** 1 Cellular Neurobiology Research Branch, Intramural Research Program (IRP), National Institute on Drug Abuse, National Institutes of Health (NIH), Department of Health and Human Services (DHHS), Baltimore, Maryland, United States of America; 2 Laboratory of Neurosciences, Intramural Research Program (IRP), National Institute on Aging, National Institutes of Health (NIH), Department of Health and Human Services (DHHS), Baltimore, Maryland, United States of America; 3 Farber Institute for Neurosciences, Thomas Jefferson University, Philadelphia, Pennsylvania, United States of America; Baylor College of Medicine, United States of America

## Abstract

**Background:**

We initiated differentiation of human embryonic stem cells (hESCs) into dopamine neurons, obtained a purified population of neuronal precursor cells by cell sorting, and determined patterns of gene transcription.

**Methodology:**

Dopaminergic differentiation of hESCs was initiated by culturing hESCs with a feeder layer of PA6 cells. Differentiating cells were then sorted to obtain a pure population of PSA-NCAM-expressing neuronal precursors, which were then analyzed for gene expression using Massive Parallel Signature Sequencing (MPSS). Individual genes as well as regions of the genome which were activated were determined.

**Principal Findings:**

A number of genes known to be involved in the specification of dopaminergic neurons, including MSX1, CDKN1C, Pitx1 and Pitx2, as well as several novel genes not previously associated with dopaminergic differentiation, were expressed. Notably, we found that a specific region of the genome located on chromosome 11p15.5 was highly activated. This region contains several genes which have previously been associated with the function of dopaminergic neurons, including the gene for tyrosine hydroxylase (TH), the rate-limiting enzyme in catecholamine biosynthesis, IGF2, and CDKN1C, which cooperates with Nurr1 in directing the differentiation of dopaminergic neurons. Other genes in this region not previously recognized as being involved in the functions of dopaminergic neurons were also activated, including H19, TSSC4, and HBG2. IGF2 and CDKN1C were also found to be highly expressed in mature human TH-positive dopamine neurons isolated from human brain samples by laser capture.

**Conclusions:**

The present data suggest that the H19-IGF2 imprinting region on chromosome 11p15.5 is involved in the process through which undifferentiated cells are specified to become neuronal precursors and/or dopaminergic neurons.

## Introduction

The potential value of human embryonic stem cells (hESCs) lies in their ability to, in theory, generate any type of human cell which might be useful either for basic research or for direct therapeutic applications such as transplantation. Methods to differentiate hESCs into multiple phenotypes including endoderm, mesoderm, and ectoderm, as well as trophoblast and germ cells, have been reported [1]–[4]. Of the many cell types that can be generated from hESCs, dopaminergic neurons of the midbrain are among the most interesting. A number of reports have described the generation of dopamine neurons from animal embryonic stem cells [5]–[9]. Dopaminergic neurons can also be generated from human embryonic stem cells, in most cases employing methods which involve co-culture with stromal cell lines such as PA6 [9]–[12].

Polysialyated neural cell adhesion molecule (PSA-NCAM) has been found to label a population of cells in the developing spinal cord, which are distinct from multipotent neuroepithelial cells and glial progenitors, and are identified as being neuronal-restricted precursor cells. These cells have the ability to develop into multiple subtypes of neurons, but generally not glia [13]. PSA-NCAM is widely used as a marker for neuronal precursors in both developing and the adult brain [14], [15]. It should, however, also be noted that at least under some circumstances a minority of PSA-NCAM positive precursor cells differentiate into astrocytes [16]. PSA-NCAM is expressed in human embryonic stem cells which are undergoing differentiation to dopaminergic neurons, at a stage prior to the expression of specific dopaminergic markers [17]. In Ntera2 human embryonal carcinoma cells undergoing dopaminergic differentiation, PSA-NCAM^+^ cells sorted by flow cytometry were found to differentiate into functional dopaminergic neurons [18]. Therefore, PSA-NCAM appears to label dopaminergic neuronal precursors developing from hESCs, in addition to other possible neuronal subtypes present in differentiating hESCs.

The purpose of the present study was to obtain a transcriptional profile of PSA-NCAM^+^ neuronal precursors derived from hESCs, which we believe includes dopaminergic neuronal precursors. Previous surveys of gene expression in developing dopaminergic neurons have employed, for example, developing brain tissue [19], [20], which includes a number of cell types, or cells from transgenic animals sorted on the basis of TH promoter activity [21]. The use of hESCs to obtain differentiating dopaminergic neurons allows for the possibility of using primary human cells, sorted to obtain a purified population, at a relatively early developmental stage. We employed PSA-NCAM as a marker for sorting differentiating hESCs to obtain a purified population of precursor cells with the potential to develop the dopaminergic neuronal phenotype. Using MPSS, which is based on sequencing of 17-mer and 20-mer tags generated by the restriction enzyme DpnII, we obtained a profile of genes expressed by developing human neurons derived from hESCs. Among the activated genes was a cluster of genes on chromosome 11p15.5, in the H19-IGF2 imprinting center, within which are the TH and IGF2 genes, and the gene for CDKN1C, or p57Kip1, which cooperates with Nurr1 to induce the dopaminergic phenotype [22].

## Materials and Methods

### hESC culture and dopaminergic differentiation of hESCs

hESC of the BG03 line (BresaGen Inc., Athens, U. S. A.) were maintained on inactivated mouse embryonic fibroblast feeder cells in medium comprised of Dulbecco's Modified Eagle's Medium/Ham's F12 supplemented with 20% knockout serum replacement, 2 mM non-essential amino acids, 2 mM L-glutamine, 50 µg/ml Penn-Strep (all from Invitrogen, Carlsbad, U. S. A.), 0.1 mM β-mercaptoethanol (Chemicon International, Inc., Temecula, U. S. A.), and 4 ng/ml of basic fibroblast growth factor (Sigma Aldrich, St. Louis, U. S. A.), as previously described [17], [23]. Cultures of hESCs were checked regularly for karyotype abnormalities. Cells were passaged by manual dissection every 4–6 days.

Dopaminergic differentiation of BG03 was induced by co-culture with the mouse stromal cell line PA6 as described previously [9]. Briefly, BG03 cells were seeded at a density of approximately 1000 clumps per 3 cm dish on a confluent layer of PA6 feeder cells in Glasgow Minimum Essential Media (Invitrogen) supplemented with 10% knockout serum replacement, 1 mM pyruvate (Sigma), 0.1 mM nonessential amino acids, and 0.1 mM β-mercaptoethanol.

### Fluorescence-activated cell sorting (FACS) and *in vitro* differentiation

Flow cytometry was used to isolate NCAM^+^ cells from the mixture of cells present in BG03 and PA6 co-cultures using single label sorting. Briefly, differentiating hESC were removed from the PA6 cell layer and dissociated with papain (Worthington Biochemical, Lakewood, U. S. A.) thoroughly washed with PBS+1 mg/mL BSA+50 µg/mL penicillin/streptomycin, filtered through a 40 µm nylon mesh cell strainer (BD Biosciences, San Jose, U. S. A.), and stained with PSA-NCAM (1 ug/million cells, Chemicon MAB1951) for 45 min in staining medium (PA6 conditioned medium, 10% FBS, and 50 µg/mL penicillin/streptomycin) at 37°C in 5% CO_2_. Cells were then washed three times with staining medium and incubated with 488-goat anti mouse IgM secondary antibody (1 µg/million cells, Invitrogen) for 45 min at 37°C in 5% CO_2_, followed by three washes with PBS+1 mg/ml BSA. Propidium iodide (1 µg/ml) was added to the cell suspension to discriminate live and dead cell populations. Cells were then filtered through a 40 µm nylon mesh cell strainer prior to sorting. The NCAM^+^ cells were sorted through a FACSstar plus cell sorter (BD Biosciences).

For *in vitro* differentiation, NCAM^+^ sorted cells were plated at 2000–4000 cells/ cm^2^ onto fibronectin-coated dishes and grown in PA6 conditioned medium with a medium change at day 1 and every two days thereafter. Following sorting, cells were allowed to differentiate for 2–3 weeks prior to immunostaining and electrophysiological recording.

### Electrophysiological recording

Cells were bathed in PBS consisting of (mM) NaCl 137, KCl, 2.68, KH_2_PO_4_ 1.47, Na_2_HPO_4_ 8.1, MgCl_2_ 0.49, CaCl_2_ 0.90, glucose 5.55, sucrose (for osmotic balance) 25.0, pH = 7.4, and were recorded in whole cell mode using patch clamp micropipettes containing (mM) KCl 140, MgCl_2_ 2, CaCl_2_ 1, EGTA 11, HEPES 10, MgATP 2, pH 7.2. Pipette resistances were 3–6 MΩ. Signals, obtained by a List EPC7 amplifier operating in either current clamp or voltage clamp mode, were digitized by a DigiData 1200 interface (Axon Instruments, Union City, U. S. A.) under command of the program pClamp v.6 (Axon Instruments). The neurotransmitters acetylcholine (100 µM), GABA (100 µM), and glutamic acid (1 mM, as the monosodium salt) were superfused by means of a microcapillary tube attached to a U-tube that was directed toward the cell under investigation and suspended above it by 200 µm. Cells were voltage clamped at −70 mV and depolarized in 10 mV steps to search for electrical excitability, and were voltage clamped at −60 mV when tested by application of neurotransmitters.

### RT-PCR and qPCR analysis

Total RNA was extracted from undifferentiated hESCs or NCAM^+^ cells using RNA STAT-60 (Tel-Test Inc., Friendswood, U. S. A.) and cDNA was synthesized by using a reverse transcription kit (RETROscript, Ambion, Austin, U. S. A.). The PCR primers are listed in Table S1. Real-time qPCR was used to quantify the levels of mRNA expression for selected genes using the DNA Engine Opticon Fluorescence Detection System (MJ Research, Waltham, U. S. A.) with DyNAmo SYBR Green qPCR kits. The content of selected genes was normalized to 18S-rRNA and standard curves were generated using 10 to 1000 ng cDNA per 20 µl reaction volume. All PCR products were checked by melting curve analysis to exclude the possibility of multiple products or incorrect product size. PCR analyses were conducted in triplicate for each sample.

### Immunocytochemistry

Immunocytochemistry and staining procedures were as described previously [24]. Briefly, cells were fixed with 2% paraformaldehyde for 30 min, blocked in blocking buffer (5% goat serum, 1% BSA, 0.1% Triton X-100) for 1 hr followed by incubation with the primary antibody at 4°C overnight. Appropriately coupled secondary antibodies (Invitrogen) were used for single and double labeling. All secondary antibodies were tested for cross reactivity and non-specific immunoreactivity. The following antibodies were used: TH (1∶1000, AF1997; R & D Systems, Minneapolis, U. S. A.), NCAM (1∶1000, Chemicon MAB1951) and TuJ1 (β-III tubulin, 1∶2000, Sigma T8660). Bis-benzamide (Hoeschst 33342, Sigma 1∶1000) was used to identify nuclei. Images were captured on either a Zeiss (Thornwood, New York, U. S. A.) or Olympus (Center Valley, U. S. A.) fluorescence microscope.

### MPSS analysis

About 20 µg of total RNA isolated from NCAM^+^ cells derived from BG03 was analyzed through an Agilent Bioanalyzer (Agilent Technologies, Santa Clara, U. S. A.), passed quality control tests and qualified for MPSS analysis. The mRNA was processed according to the MPSS protocol as outlined previously [25], [26] with some modifications. Briefly, the mRNA was reverse-transcribed and the cDNA was digested with Dpn II. The cDNA of the last Dpn II site and the downstream 16 bases were cloned into a Megaclone vector. The resulting library was amplified and loaded onto microbeads. About 1.2 million microbeads were loaded into each flow cell and the signature sequences were determined by a series of enzymatic reactions as outlined in the above publications. The abundance for each signature was converted to transcripts per million (tpm) for the purpose of comparison between samples. To generate a complete, annotated human signature database, we extracted all the possible signatures from the human genome sequence and the human UniGene sequences. Each virtual signature is ranked and assigned a class, based on its position and orientation in the original sequence. The annotation database is established based on the virtual signatures, their classes and their corresponding genes so that each signature only has one corresponding annotation. The database was then used to annotate the data from the experiments.

### Analysis of human postmortem brain neurons

Fresh frozen tissue blocks corresponding to the substantia nigra (SN) from 4 control and 4 Parkinson's disease patients older that 55 years of age were obtained from the New York Brain Bank at Columbia University and the Brain Institute of University of Florida (information on the cases used is shown in Table 1). Fresh frozen sections containing neuromelanin positive cells were cut 8 µm thick, briefly fixed in 70% EtOH and briefly stained with Nissl stain for neuronal identification using the HistoGene®LCM frozen section staining kit (Molecular Devices, Sunnyvale, U. S. A.) to avoid RNA degradation. Before initiating microdissection, we determined RNA integrity by detecting 18S and 28S bands on the RNA 6000 Nano LabChip (Agilent Technologies) from extracted total RNA. The rRNA ratios ranged between 0.7–1.3. Approximately 200 neuromelanin positive neurons were microdissected with a Leica LSMLD instrument. Total RNA was isolated from the neuronal samples with the Pico-Pure RNA isolation kit (Arcturus Engineering, Mountain View, U. S. A.). A double-round amplification procedure was performed with the HS RiboAmp RNA amplification kit, according to manufacturer's instructions (Arcturus Engineering). We assessed the amplified copy RNA quality with the RNA 6000Nano LabChip (Agilent Technologies) and its quantity by UV spectrophotometry at 260 nm wavelength.

**Table 1:** Control and Parkinson's disease case information.

Case	Age	Sex	PM1 (hours)	Cause of Death
Parkinson's disease cases				
1	80	F	Unknown	Unknown
2	76	M	18	Pneumonia
3	81	F	17	Complication of disorder
4	79	F	14	Unknown
Aged matched control cases				
5	82	F	9	Unknown
6	71	M	Unknown	Cancer
7	77	M	18	Pancreatic cancer
8	80	M	21	CPA
9	86	M	Unknown	Congestive heart failure

For cDNA synthesis, 0.5 µg copy RNA or 5 µg total RNA (human SN total RNA, Ambion) was mixed with 2 µl random decamer primers (Ambion) and 1 µl of a 10 mM dNTP mix (Roche Applied Science, Indianapolis, U. S. A.), filled up to a final volume of 13 µl and incubated for 10 min at 65°C. After cooling on ice, 7 µl of RT master mix (SuperScript III Reverse Transcriptase, Invitrogen) were added and cDNA synthesis was performed at 50°C for 60 min and terminated by incubating for 15 min at 75°C. PCR grade water was added to the reaction mixture to a final volume of 100 µl. PCR was performed on the ABIHT7900 instrument (Applied Biosystems [ABI], Foster City, U. S. A.), using the DNA binding dye SYBR Green I for the detection of PCR products. PCR reactions were set using 2 µl cDNA with 18 µl of a SYBR Green I master mix containing forward and reverse primers (Table S2), MgCl2 and SYBR Green. A blast search for each primer pair verified that the binding sites were unique for each gene studied. For detection of TH in the collected samples, a pre-designed assay-on-Demand^TM^ Taqman^TM^ probe and primer pair was obtained from ABI (Mm00447546_m1).

## Results

**Figure 1:**
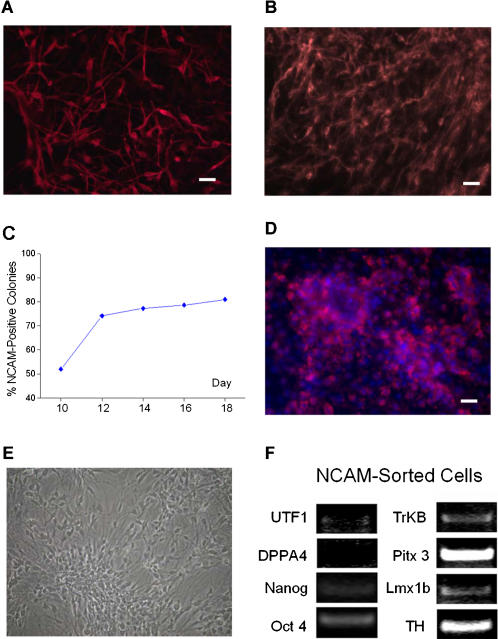
Expression of neuronal markers in hESCs during dopaminergic differentiation and in FACS-isolated NCAM^+^ cells. (A) A culture of hESCs differentiated for 16 days on PA6 cells immunostained for β-III-tubulin. (B) Immunostaining for TH after 21 days. Bar = 50 µm. (C) Quantification of hESC colonies positive for PSA-NCAM, during differentiation of hESCs co-cultured with PA6 cells. (D) Expression of PSA-NCAM in day 14 hESCs by immunocytochemistry. Bar = 50 µm. (E) Morphology of FACS-isolated hESC by PSA-NCAM after 14 days of subsequent differentiation. (F) Expression of markers for undifferentiated hESC and neuronal markers in FACS-isolated NCAM^+^ cells by RT-PCR.

On day 0, undifferentiated BG03 cells were transferred to co-culture with mouse stromal PA6 cells. TH-positive cells were first detected in small numbers at days 10–12. Numerous β-III tubulin (Tuj1)^+^ cells and TH^+^ cells were identified in the cultures by days 16 and 21, respectively (Fig. 1*A–B*).

PSA-NCAM was expressed in a typical cell surface pattern in 52% of the hESC colonies at day 10, and the percentage of positive colonies increased to 75% at day 12 (Fig. 1*C*). No significant increase in PSA-NCAM^+^ cells was observed between days 12–18 of differentiation. A high percentage of cells (>50%) in the majority of colonies (>50%) were positive for NCAM (Fig. 1*D*).

Based on the time course of NCAM and TH expression during differentiation, we chose day 14 as the time point for enriching dopaminergic neuronal precursors by FACS. The yield of PSA-NCAM^+^ cells by FACS was approximately 21% of the total cells.

### NCAM^+^ cells differentiate into TH^+^ neurons *in vitro*

When FACS-isolated PSA-NCAM^+^ cells were placed back into culture subsequent to sorting, cells resembling neural stem cells with rosette structures, and cells with a neuron-like bipolar morphology were present (Fig. 1*E*).

**Figure 2:**
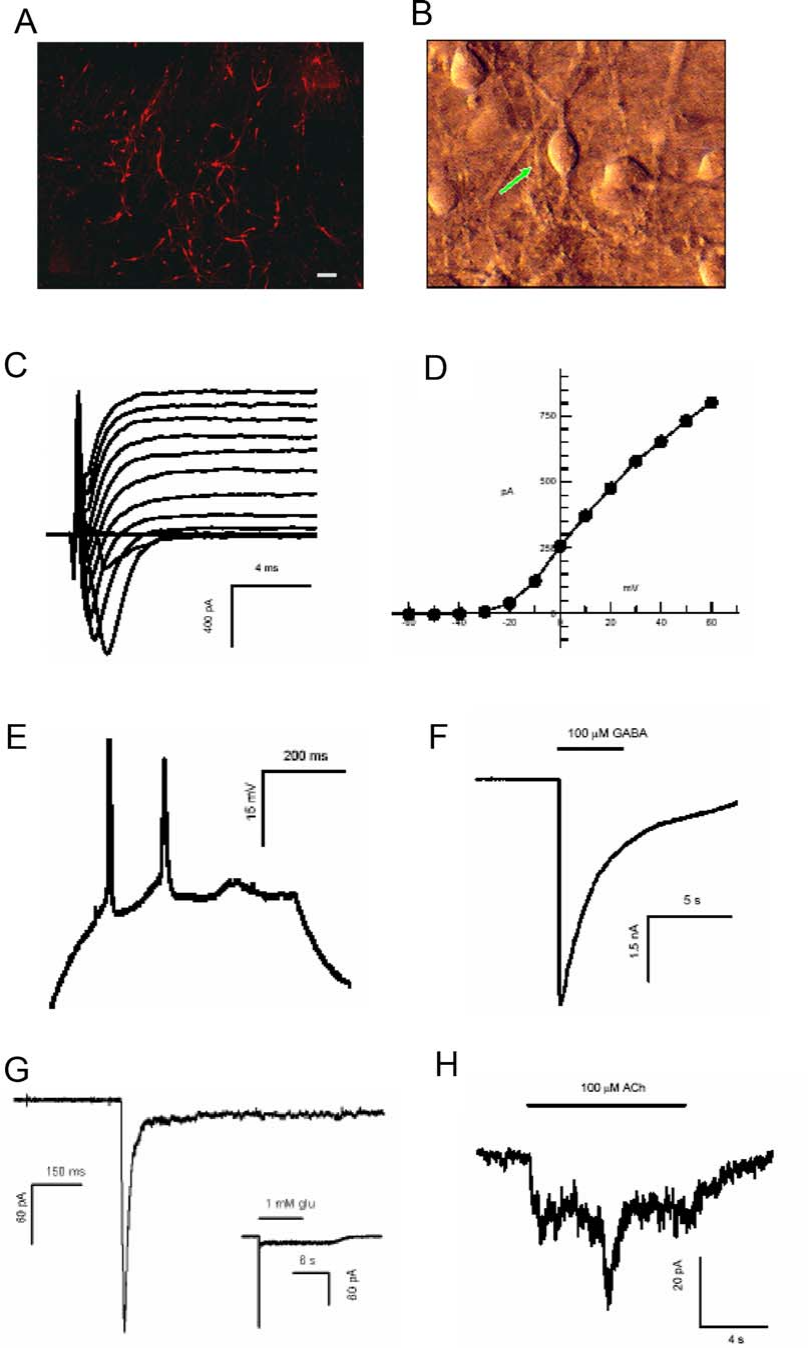
FACS-isolated NCAM^+^ cells derived from hESCs differentiated into dopaminergic neurons sorted *in vitro* NCAM^+^. Cells were differentiated in medium conditioned on PA6 cells for 21 days prior to immunocytochemical staining and electrophysiological recording. (A) Representative TH staining (Calibration bar = 100 µm). (B) A representative PSA-NCAM sorted cell. (C–D) The downward deflections are inward currents mediated by sodium channels. They are followed by the prolonged upward currents, the delayed rectifier, mediated by potassium channels. (E) Action potentials trigged by a step injection of current in the cell under the current clamp mode of recording. (F–H) Responses to neurotransmitters GABA (F), Glutamate (G) and acetylcholine (H).

To evaluate the degree of differentiation in NCAM^+^ cells, we determined the expression of several undifferentiated hESC markers and dopaminergic/neuronal markers by RT-PCR. No or very low levels of expression of three undifferentiated markers (UTF1, DPPA5 and Nanog) was found in PSA-NCAM^+^ cells, whereas Oct4 was detected (Fig. 1*F*). Expression of four neuronal markers (TH, Lmx1b, Pitx3 and TrkB) was detected in sorted cells (Fig. 1*F*). Numerous TH-positive cells were present after 10 to 20 days of culture in the presence of SHH and FGF8 (Fig. 2*A*).

Electrophysiological recording from FACS-sorted PSA-NCAM^+^ cells that were subsequently differentiated in PA6 conditioned medium for three weeks showed that these cells were electrically excitable (Fig. 2*B*), as evidenced by inward sodium currents (Fig. 2*C*) and by the delayed rectifier currents (carried by potassium) seen as the upward bend in the current-voltage plot (Fig. 2*D*). Of 44 cells tested, 30 (68%) gave clear sodium currents, and 32 (73%) displayed the characteristic delayed rectifier curve. Action potentials were observed in many cells recorded under current clamp conditions (Fig. 2*E*). In addition, sensitivity to the neurotransmitters GABA, glutamate and acetylcholine was tested. Most cells responded to GABA (20/28 or 71%) and generally gave very large currents of hundreds of pA (Fig. 2*F*). A smaller proportion of cells responded to glutamate (12/29 or 41%) and acetylcholine (5/27 or 18%), with smaller currents of 100 pA or less (Fig. 2*G–H*).

### MPSS profiling

The total number of distinct sequences detected by MPSS was 11912. The complete datasets for both 17-nucleotide and 20-nucleotide signatures are available for download from ftp://137.187.144.38/freed. The distribution frequency of these signatures is shown in Table 2, with the signature distribution in undifferentiated hESCs shown for comparison. The transcriptional complexity of hESC-derived NCAM^+^ cells (11912 total signatures) was substantially less than that of undifferentiated hESCs (38403 total signatures), but comparable to that seen in hESC-derived EBs [27] and neural stem cells (unpublished data). Similar to undifferentiated hESCs, most genes were expressed at relatively low levels with greater than 60% of the genes being expressed at lower than 50 tpm and 25% lower than 10 tpm.

Fewer than 2% of the expressed genes (131 signatures) were expressed at a level higher than 1000 tpm. Only 8 genes were expressed at greater than 10,000 tpm, and only two, IGF2 and H19, were expressed at greater than 50,000 tpm. Most of the highly abundant genes were ribosomal, mitochondrial and housekeeping genes, whereas growth factors, transcription factors and regulators of gene expression were generally expressed at lower tpm. A list of the 100 most highly expressed genes is shown in Table 3, which also lists the 100 genes most highly expressed in undifferentiated hESCs.

**Table 2:** TPM distribution in NCAM^+^ cells and undifferentiated hESCs.

NCAM^+^			Undifferentiated hESCs		
MPSS (tpm)	No. of Signatures	Percentage	MPSS (tpm)	No. of Signatures	Percentage
≥10,000	8	0.11	≥10,000	2	0.02
≥5,000	19	0.28	≥5,000	16	0.20
≥1,000	131	1.92	≥1,000	122	1.52
≥500	266	3.91	≥500	279	3.48
≥100	1555	22.84	≥100	1621	20.22
≥50	2708	39.78	≥50	2953	36.83
≥10	5088	74.74	≥10	6345	79.13
≥3	6352	93.30	≥3	7761	96.79
≥1	6808	100.00	≥1	8018	100.00

**Table 3:** The 100 most-highly expressed genes (in TPM) for NCAM^+^ and undifferentiated BG02 cells.

Category	Only in NCAM+ Cells	Common Genes	Only in BG02 Cells
Ribosomal proteins	RPL17, RPL7A, RPS18, RPS27, RPS5 (5)	PRDX6, RPL10, RPL11, RPL13, RPL13A, RPL23A, RPL26, RPL28, RPL30, RPL32, RPL35, RPL37A, RPL4, RPL6, RPL8, RPS15, RPS15A, RPS17, RPS19, RPS2, RPS20, RPS27A, RPS28, RPS3, RPS3A, RPS7, RPSA (27)	FAU, RPL12, RPL14, RPL21, RPL22, RPL27A, RPL5, RPS6, RPS9 (9)
Mitochondrial protein	ATP5B (1)	ATP5O (1)	NDUFS5 (1)
Structural proteins	COL18A1, KRT18, KRT19, TPM1 (4)	ACTB, CFL1 (2)	DMD, MRCL3, PFN1, STMN1, TMSB4X, VCP (6)
Prime membrane proteins and ECM proteins	AQP1, CD99, COL1A1, COL1A2, COL3A1, COL5A1, COL6A3, DCN, FBLN1, FN1, LUM, SLC2A1 (12)	COL6A1, KRT8 (2)	CD24, GJA1, IFITM1 (3)
Metabolic proteins	ARF1, EEF2, GPX4, GSTP1, ISYNA1, OAZ1, P4HB, PLTP, SERPINH1, TPI1, UBA52, UBB, UPF2 (13)	ALDOA, EEF1A1, FTH1, FTL, GAPD, PPIA, SDHB, SUI1 (8)	EEF1G, HSPCA, PKM2, PSMA7, SSB, UCHL1, UGP2 (7)
Signal transduction proteins	BST2, CCNI, CDKN1C, FRZB, IGF2, MAGED2, NGFRAP1, S100A10, SPARC (9)	GNAS, HSPCB, MIF, SERF2, TMSB10, TPT1 (6)	DCOHM, GDI2, GNB2L1, HSPE1, YWHAQ, YWHAE (6)
Nuclear proteins	HAND1, JUND, NSEP1, PITX1 (4)	H3F3A (1)	CLU, DNMT3B, G22P1, HMGA1, HNRPA1, IGAAD, LIN28, NACA, NASP, NCL, NPM1, NSEP1, POU5F1, PTBP1, PAI-RBP1, SET, TERF1, XRCC5 (18)
Unknown genes	C20orf149, LOC92154, MGC5178, NME4, syntaxin 7 (5)		Hs.461412, Hs.476965, Hs.571693 (3)
Total Number:	53	47	53

### Genes uniquely expressed in NCAM^+^ cells

Of the 11912 expressed genes detected in NCAM^+^ cells, 232 were either expressed only in NCAM^+^ cells but not in undifferentiated hESCs, or expressed at levels at least 10-fold higher in NCAM^+^ cells as compared to undifferentiated hESCs. These included 195 known genes and 37 novel genes. Of the 195 known genes, there were 66 that encoded signal transduction proteins, 46 ECM proteins, 37 nuclear proteins, 33 metabolic proteins, 10 structural proteins, 2 ribosomal proteins and one mitochondrial protein (Table S3). Notably, several transcription factors associated with early neural and dopaminergic differentiation were highly expressed in NCAM^+^ cells. For example, Msx1 was found to be expressed at a very high level (1098 tpm) in NCAM^+^ cells but was absent in undifferentiated hESCs. Other transcription factors that were highly expressed in NCAM^+^ cells included Pitx1 and Pitx2. We also noted that many genes of the solute carrier family were expressed at very high levels in NCAM^+^ cells (Table 4). In contrast, several markers characteristic of pluripotency including Oct4 and Nanog were not detected in NCAM^+^ cells.

**Table 4:** Genes of the solute carrier family which were highly expressed in NCAM^+^ cells.

Accession	Symbol	Description	NCAM+	BG02
Hs.473721	SLC2A1	Solute carrier family 2 (facilitated glucose transporter), member 1	2592	101
Hs.529285	SLC40A1	Solute carrier family 40 (iron-regulated transporter), member 1	498	0
Hs.513147	SLC7A7	Solute carrier family 7 (cationic amino acid transporter, y+ system), member 7	264	0
Hs.396783	SLC9A3R1	Solute carrier family 9 (sodium/hydrogen exchanger), member 3 regulator 1	210	0
Hs.403790	SLC25A28	Solute carrier family 25, member 28	200	0
Hs.521934	SLC39A4	Solute carrier family 39 (zinc transporter), member 4	183	0
Hs.500761	SLC16A3	Solute carrier family 16 (monocarboxylic acid transporters), member 3	176	0
Hs.363138	SLC27A1	Solute carrier family 27 (fatty acid transporter), member 1	150	0

**Figure 3:**
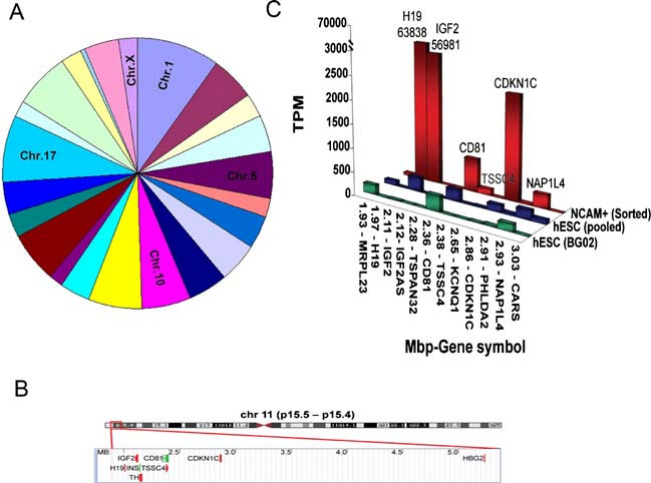
Chromosomal distribution of genes in NCAM^+^ sorted cells mapped to chromosomes. (A) Plot of percentages of total detected genes expressed on each chromosome. (B) Representation of the locations of the highly-expressed genes in 11p15.5, as described in the text. (C) Expression levels (tpm) for genes in the 11p15.5 region in NCAM^+^ neuronal precursor cells as compared to undifferentiated hESCs.

When the 232 uniquely or highly expressed in NCAM^+^ cells were sorted for chromosomal locations, they were generally distributed across all chromosomes, with relatively higher numbers of genes on chromosomes 1, 5, 10, 11, 14, 17, and 19 (Fig. 3*A*). Examination of cytogenetic map locations (Table S4) revealed a number of localized clusters of genes that were highly expressed in NCAM^+^ cells; most notably, five genes located on chromosome 11p15.5, at or near the H19-IGF2 imprinting center were highly expressed. These were H19, IGF2, CDKN1C, TSSC4, and HBG2. Representations of locations and expression levels of genes in this chromosomal region, from 1.9 to 5.5 MB from the telomere, in NCAM^+^ cells as compared to undifferentiated hESCs are shown in Figure 3*B and 3C*. It is clear that several genes in this region are highly expressed in NCAM^+^ sorted cells, but not in undifferentiated hESCs (Fig. 3*C*). Among the genes in this region are H19 and IGF2, the only two genes with expression above 50,000 tpm. Some of the same genes are expressed in embryoid bodies and oligodendrocyte precursors as well, although not at the high levels seen for NCAM^+^ sorted cells (data not shown). There was also a prominent cluster of highly expressed genes at 14q24.3 (see Table S4).

Remarkably, the gene for TH, a marker of mature dopaminergic neurons, and the rate-limiting enzyme in catecholamine biosynthesis, is located within the 11p15.5 gene cluster, and is known to be expressed in the sorted cell population (Fig. 1*F* and 2*A*). Although TH was not detected in the NCAM^+^ cells, this is likely because TH is not reliably detected by the classic MPSS protocol where amplification of cDNA between the last Dpn II site and the polyA tail is required. In rare cases, where there is a long span of more than 1 kb between the polyA tail and the first DpnII site, these signatures usually fail to be amplified and are not detected. TH was also not detected in a MPSS database of 32 human tissues which included the adrenal gland ([28]; http://mpss.licr.org/index.php).

### Verification of gene expression

**Figure 4:**
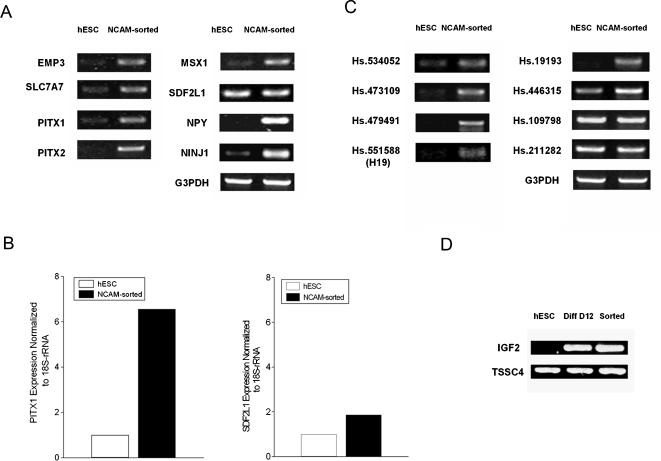
Verification of genes uniquely expressed in NCAM^+^ cells by RT-PCR and qPCR. Eighteen genes that were highly expressed in NCAM^+^ cells were selected for verification by RT-PCR. (A) Expression of EMP3, SLC7A7, Pitx1, Msx1, Pitx2, SDF2L1, NPY and NINJ1 was detected in NCAM^+^ cells by RT-PCR. Of these, two (SDF2L1 and NINJ1) were also expressed in undifferentiated cells, and the other 6 genes (EMP3, SLC7A7, Pitx1, Pitx2, Msx1 and NPY) were expressed only in NCAM^+^ cells but were absent or expressed at very low levels in undifferentiated hESCs. (B) qPCR results for Pitx1 and Sdf2l1 expression in NCAM^+^ cells and undifferentiated hESCs. (C) Expression of seven unknown genes lacking known protein products in undifferentiated hESCs and in NCAM^+^ sorted cells. Hs.534052, Hs.473109, Hs.479491, Hs.19193 and Hs.551588 (H19) were detected only in NCAM^+^ sorted cells. Hs.446315 was detected in undifferentiated hESC, but at a lower level than for NCAM^+^ sorted cells. No difference in expression was detected for Hs.109798 or Hs.211282. (D) RT-PCR examination of IGF2 and TSSC4 expression in undifferentiated hESCs and NCAM^+^ sorted cells IGF2 and TSSC4 were also examined in 12-day differentiated, unsorted cultures (Diff D12). IGF2 was not detected in the undifferentiated hESCs, whereas no difference was seen for TSSC4.

To verify the differential expression in NCAM^+^ cells detected by MPSS, we selected 18 genes (EMP3, SLC7A7, Pitx1, Msx1, Pitx2, SDF2L1, NPY and NINJ1, Hs.551588 (H19), Hs.19193, Hs.473109, Hs.109798, Hs.534052, Hs.446315, Hs.211282 and Hs.479491, TSSC4, and IGF2) for RT-PCR analysis (Fig. 4).

Expression of all genes was detected in NCAM-sorted cells by RT-PCR, indicating the reliability of MPSS analysis. Of the known genes, two (SDF2L1 and NINJ1) were also expressed in undifferentiated cells whereas the other six (EMP3, SLC7A7, Pitx1, Pitx2, Msx1, and NPY) were absent or only slightly expressed in undifferentiated hESCs (Fig. 4*A*).

Among the unknown genes, Hs.446315, Hs.109798, and Hs.211282 were detected in both NCAM^+^ cells and undifferentiated hESCs (Fig. 4*B*). Hs.534052, Hs.473109, Hs.479491, Hs.19193 and Hs.551588 (H19) were undetectable or only weakly expressed in undifferentiated hESCs, but strongly expressed in NCAM-sorted cells. These differentially-expressed genes might be further explored as markers for the dopaminergic phenotype.

We selected 2 genes (Pitx1 and SDF2L1) for qPCR analysis. As shown in Figure 4*B*, Pitx1 and SDF2L1 were expressed at 6.6-fold and 1.8-fold higher in NCAM^+^ cells than in undifferentiated hESCs, respectively.

Three genes in the 11p15.5 region were selected for RT-PCR analysis. Hs.551588 (H19, Fig. 4*B*) and IGF2 were expressed in NCAM^+^ sorted cells, but not undifferentiated hESCs (Fig 4*D*). TSSC4 was expressed in undifferentiated hESCs as well. Expression of IGF2 and TSSC4 was also detected in unsorted 12-day differentiated hESCs (Fig. 4*D*).

### Expression of H19, IGF2, and CDKN1C in human dopaminergic neurons

**Figure 5:**
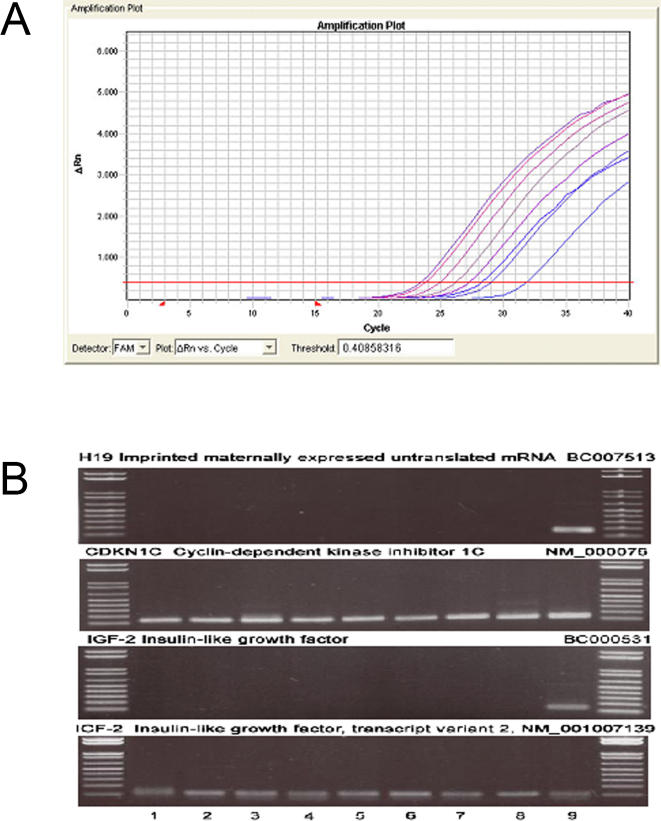
Expression of H19, CDKNIC and 1GF1 in mature human dopaminergic neurons. (A) Amplification plot for tyrosine hydroxylase copy RNA from neuromelanin-positive cells collected by laser capture microdissection in 4 control and 4 Parkinson disease cases (samples 1–8 in Fig. 2). For all samples analyzed, a sequence specific for tyrosine hydroxylase (Ct 22-32) was amplified, indicating the selection of dopaminergic cells. (B) Images of ethidium bromide gels showing the PCR products amplified from reversed-transcribed RNA extracted from the substantia nigra. Lines 1–8 correspond to amplified copy RNA from neuromelanin (+) neurons collected by laser capture microdissection from Parkinson's disease patients (1–4) and age-matched controls (5–8). Line 9 corresponds to total RNA isolated from the substantia nigra of an age-matched control. DNA size markers (1 kb plus ladder, Life tech) are shown on left and right.

We also examined expression of H19, IGF2, and CDKN1C in laser-captured dopamine neurons, identified on the basis of neuromelanin presence, from a series of human postmortem RNA samples from human cases of Parkinson's disease and controls (see Table 1). TH was amplified from each of the samples, verifying that the collected neurons were dopaminergic (Fig. 5*A*). H19 was found to be expressed in the midbrain, but was not expressed in dopamine neurons per se. CDKN1C was expressed in all samples of laser-captured dopamine neurons (Fig. 5*B*). These samples were probed for two IGF2 transcripts, and one was positive (NM_001007139), while BC000531 was negative (Fig. 5*B*). Therefore, certain genes within the H19 cluster, including TH, CDKN1C, and one IGF2 transcript are expressed in mature human dopamine neurons *in vivo*.

## Discussion

The chromosomal region 11p15.5 has been extensively studied, largely because of it's properties as an imprinting center [29]–[31]. This region contains a number of genes which are imprinted, that is, expressed only from either the paternal or maternal allele, as well as several other non-imprinted genes [32]–[34]. In addition to the two widely-studied imprinted genes, H19 which is maternally imprinted and codes for an un-translated RNA, and the IGF2 gene, which is imprinted paternally, this region contains at least ten other imprinted genes [29]. H19 and IGF2 are expressed in various tissues during development and down-regulated in most mature tissues [35]. H19 expression can be up-regulated in tumors, but is also expressed in certain normal mature tissues, notably adrenal gland, muscle, heart, thyroid, mammary gland, skin, and esophagus; nonetheless, the function of H19 remains unclear [36]. The IGF2 gene, in contrast, has a number of transcripts, including an insulin-IGF2 read-through transcript and an antisense transcript [37], some of which are widely expressed in mature tissues.

Although the H19 region has been studied mainly in association with imprinting, it appears that this region contains a precisely-structured cluster of genes which evolutionarily precedes imprinting. An almost identical gene grouping is present in the chicken [38], where no imprinting occurs. Also, a paralagous grouping of genes is present on chromosome 12, apparently haven arisen through gene duplication and subsequent tetraploidy [39]. A similar imprinting center, which contains an almost identical grouping of genes but with major differences in both imprinting and deletion of large repetitive sequences, occurs on chromosome 7 of the mouse [32]. The mouse H19-IGF2 region on chromosome 7 also regulates expression of genes on mouse chromosome 11 via CCCTC-binding factor [40]. In addition, the 11p15.5 region influences gene expression on chromosome 17, in an area which contains a number of genes related to neuronal function, including NF1 [41], the dopamine D3 receptor [42], and mSWiP-2 or WSB-1, a SHH interacting protein [43], [44]. Therefore, it seems probable that the H19-IGF2 imprinting center contains a clustering of genes which is of fundamental biological importance, beyond their association for the purpose of imprinting. In fact, it might be conjectured that imprinting arose subsequent to evolution of the cluster, as a mechanism to control levels of gene expression in a region of major developmental significance. Through many lines of evidence, it has been recognized that genes in this region are important for development [e.g., 45]–[47].

Despite the extensive attention which has been paid to this gene cluster, no specific biological process which might be related to this grouping of genes has been identified. It has recently been reported that H19 is a microRNA precursor, and may regulate development by that means [48]. It is perhaps noteworthy that this region contains two genes which are of central importance to major endocrine and secretory functions, the insulin gene and the TH gene, encoding the rate-limiting enzyme in catecholamine synthesis. TH is crucial for the synthesis of norephinephine and epinephrine, which are produced mainly in the adrenal medulla. In addition, the TH enzyme is rate-limiting for catecholamine synthesis in central norephinephrine and dopamine-producing neurons; dopaminergic neurons are present and play important roles in the control of motor function in virtually all animal species [e.g., 49].

The occurrence of these two genes within the H19-IGF2 imprinting center is suggestive, yet there is no apparent reason for their proximity or grouping within other genes in this region. It is noteworthy, however, that imprinting of genes in this region appears to have a role in the regulation of insulin secretion [50], [51]. H19 and IGF2 are expressed in the adult pancreas [52]. The present data suggest that the H19-IGF2 region may be involved in dopamine neuron specification as well.

It is recognized that clusters of co-expressed genes are distributed throughout the genome [53]. In hESCs and EBs, regions of chromosomal co-expression have been identified, including an area near the telomere of chromosome 11p [54] which would include H19 and IGF2. Expression of H19 and IGF2 is generally high during embryogenesis, but in mature tissues H19 shows a very restricted expression. De-differentiation, as occurs in tumor cells, generally results in a re-expression of H19 in various cell types [47]. Although H19 and IGF2 are expressed in the adult pancreas [52], there is not a simple association between H19 and IGF2 expression with the expression of TH or insulin. In the developing adrenal gland, H19 is strongly expressed in the cortex, while IGF2 is highly expressed in the fetal zone. The fetal zone contains the precursors of chromaffin cells, which later aggregate and give rise to the medulla, where high levels of TH are expressed. Interestingly, in an MPSS study of gene expression in adult mouse tissues, the adult adrenal gland had high levels of expression of TH (2472 tpm), and even higher expression of CD81 (3701 tpm) and CDKN1C (4070 tpm), while H19 and IGF2 were expressed at relatively low levels (164 and 23 tpm respectively; mouse Reference Transcriptome Database (MRTD), http://www.ncbi.nlm.nih.gov/projects/geo/info/mouse-trans.html).

Dopaminergic neurons perform a wide range of functions related to their ability to form and maintain connections with other neurons, interact with other cells, process and release neurotransmitter, and adapt to environmental signals and stressors [55]–[58]. Understanding the unique features of dopaminergic neurons is likely to be important for enabling the design of therapeutic and neuroprotective strategies, as well as to manipulate their function for the purpose of targeting human disorders. The process by which midbrain dopaminergic differentiation initiated in mice is reasonably well understood. Glia-like floor plate cells are converted to dopaminergic neurons through the actions of SHH and FGF8. SHH regulates the expression of a series of homeodomain transcription factors including MSX1 and Lmx1a, which initiate the specification of the dopaminergic neuronal phenotype [59]. Subsequent differentiation is controlled by additional transcription factors, especially Nurr1, Lmx1b, En1, Pitx3, and Ngn2 [8], [60], [61]. Nurr1 cooperates with CDKN1c, or p57Kip1, to induce the dopaminergic phenotype [22].

The mature phenotype of dopaminergic neurons is known to involve a number of features related to neurotransmitter production and release, as well as a number of less-well understood properties related to the maintenance of synaptic connections. Therefore, the process through which the dopamine neuronal phenotype is initiated, and the final mature functional phenotype of dopamine neurons have been studied in some detail. The intermediate steps in the maturation of dopaminergic neurons involve a complex process, many elements of which are still unknown.

Overall, in addition to TH, the 11p15.5 region contains a number of genes which are known to be important in the function of the brain dopaminergic system, including CDKN1C, the D4 dopamine receptor, and CD81 [62]–[64]. In addition, the CDKN1C gene, which is also within the 11p15.5 cluster, is important for differentiation of dopaminergic neurons [22], in addition to a more general role in cell cycle exit and cell differentiation [65].

Despite many studies involving genetic manipulation of the H19 locus and examination of expression patterns, the function of the H19 gene and the surrounding region remains uncertain. Bendall and coworkers [66] have recently reported that IGF2 is involved in maintaining hESC self-renewal and pluripotency. The present data suggest that the H19-IGF2 imprinting region on chromosome 11p15.5 is involved in regulation of the process through which undifferentiated cells are specified to become dopaminergic neuronal precursors.

## Supporting Information

Table S1 Characteristics of primers utilized for hESC samples
(0.06 MB DOC)

Table S2 Characteristics of primers used for human brain samples
(0.03 MB DOC)

Table S3 Genes uniquely expressed in NCAM+ cells
(0.43 MB DOC)

Table S4 Cytogentic map locations for highly-expressed genes in PSA-NCAM+ FACS-sorted cells
(0.29 MB DOC)
