# Supplementary material for: Gene Expression Profile of Neuronal Progenitor Cells Derived from hESCs: Activation of Chromosome 11p15.5 and Comparison to Human Dopaminergic Neurons
Source: PLoS One. 2008 Jan 9;3(1):e1422. doi: 10.1371/journal.pone.0001422 (PMC2170519; doi:10.1371/journal.pone.0001422)
Supplement: Table S1 — Characteristics of primers utilized for hESC samples (0.06 MB DOC) [file pone.0001422.s001.doc]

Table S1. Characteristics of primers utilized for hESC samples

| **EMP3** (55oC) Hs. 9999 | | |
| --- | --- | --- |
| 161bp | forward primer (20-mer) | 264 tctcagcccttcacatcctc 283 |
|  | reverse primer (20-mer) | 424 gccattctcgctgacattac 405 |
| **SLC7A7** (53oC) Hs. 513147 | | |
| 191bp | forward primer (21-mer) | 32 ttggaagatggaggagagatg 52 |
|  | reverse primer (22-mer) | 222 gtaagttcaaaggttgaagctg 201 |
| **PITX1** (50oC) Hs.84136 | | |
| 139bp | forward primer (19-mer) | 189 cgggttggttttgtgtttg 207 |
|  | reverse primer (19-mer) | 327 ccatcccgagagaaattgc 309 |
| **MSX1** (54oC) Hs. 424414 | | |
| 213bp | forward primer (19-mer) | 94 ttaaccctcacactgctcc 112 |
|  | reverse primer (21-mer) | 306 ctctagctctgttcaactgtc 286 |
| **PITX2** (56oC) Hs. 92282 | | |
| 296bp | forward primer (18-mer) | 1517 aaagcaaagcagcactcc 1534 |
|  | reverse primer (21-mer) | 1812 gaaagatgtcagacactgagg 1792 |
| **SDF2L1** (54oC) Hs. 303116 | | |
| 113bp | forward primer (21-mer) | 660 cagtgccaacacgcacaatac 680 |
|  | reverse primer (22-mer) | 772 atccacccatccatccacacac 751 |
| **NINJ1** (55oC) Hs. 494457 | | |
| 164bp | forward primer (20-mer) | 317 cctcatctccatctcccttg 336 |
|  | reverse primer (22-mer) | 480 tgatgaagatgttgactaccac 459 |
| **NPY** (53oC) Hs. 1832 | | |
| 154bp | forward primer (21-mer) | 243 cgacactacatcaacctcatc 263 |
|  | reverse primer (21-mer) | 396 caagtctcatttcccatcacc 376 |
| **IGF2** (55oC) Hs. 549043 | | |
| 164bp | forward primer (20-mer) | 210 cctttcacgttcactctgtc 229 |
|  | reverse primer (22-mer) | 373 gagaattcgtctgattgtccag 352 |
| **TSSC4** (53oC) Hs. 523424 | | |
| 198bp | forward primer (20-mer) | 1 gttgagcagctgaacagagg 20 |
|  | reverse primer (20-mer) | 198 gttcctgcctcagccatgcg 178 |

Unknown Genes

| **C110rf9** (56oC) Hs.473109 | | |
| --- | --- | --- |
| 197 bp | forward primer (21-mer) | 971 ggaaggaatggaaggatggag 991 |
|  | reverse primer (20-mer) | 1167 accaggcaccactaagaaac 1148 |
| **C60rf48** (54oC) HS.109798 | | |
| 263 bp | forward primer (19-mer) | 318 agggacaatgcaccttcac 336 |
|  | reverse primer (21-mer) | 580 gctgctcacacaggataatac 560 |
| **ZFP36** (53oC) HS.534052 | | |
| 137 bp | forward primer (19-mer) | 1174 attaacccactcccctgac 1192 |
|  | reverse primer (22-mer) | 1310 tcattgcctcccttaaatatgc 1289 |
| **C10orf5** (53oC) HS.446315 | | |
| 263 bp | forward primer (22-mer) | 571 gtgacattccagttccaagaag 592 |
|  | reverse primer (19-mer) | 833 agagaaatcctgactgccc 815 |
| **MGC11256** (56oC) HS.211282 | | |
| 224 bp | forward primer (20-mer) | 813 tgtaagaacgccaacggctc 832 |
|  | reverse primer (21-mer) | 1036 tcaggacacacacagacgtag 1016 |
| **FLJ23091** (54oC) HS.479491 | | |
| 277 bp | forward primer (20-mer) | 755 ttcacatctcccaagactcc 774 |
|  | reverse primer (20-mer) | 1031 tcctcctccaataccacacc 1012 |
| **H19** (55oC) HS.551588 | | |
| 210 bp | forward primer (19-mer) | 686 tgccattcaagccgttatc 704 |
|  | reverse primer (19-mer) | 895 accagttcaaccatctgcc 877 |
| **?** (53oC) HS.19193 | | |
| 182 bp | forward primer (20-mer) | 985 atgctccctccttcctcaag 1004 |
|  | reverse primer (19-mer) | 1166 tcctccacctcaacaaacc 1148 |
