# Supplementary material for: Gene Expression Profile of Neuronal Progenitor Cells Derived from hESCs: Activation of Chromosome 11p15.5 and Comparison to Human Dopaminergic Neurons
Source: PLoS One. 2008 Jan 9;3(1):e1422. doi: 10.1371/journal.pone.0001422 (PMC2170519; doi:10.1371/journal.pone.0001422)
Supplement: Table S2 — Characteristics of primers used for human brain samples (0.03 MB DOC) [file pone.0001422.s002.doc]

Table S2. Characteristics of primers used for human brain samples

| Gene | Accession | Primer Pair Sequence | Product Size |
| --- | --- | --- | --- |
| H19 | BC007513 | F: 5’-ccc acc acc tcc ctc ttc -3’  R: 5’- cac act cac gca cac tcg -3’ | 202 bp |
| IGF2 | BC000531 | F: 5’- att gct cta ccc acc caa g -3’  R: 5’- gga tgg aac ctg atg gaa ac -3’ | 152 bp |
| IGF2 | NM_001007139 | F: 5’- ctg cca tca cct gaa gac c - 3’  R: 5’ - tta gcg tta aag gag ttg agt tg -3’ | 109 bp |
| CDKNIC | NM_000076 | F: 5’- tgg gac cgt tca tgt agc -3’  R: 5’- gga cca gtg tac ctt ctc g -3’ | 146 bp |
