# Supplementary material for: Gene Expression Profile of Neuronal Progenitor Cells Derived from hESCs: Activation of Chromosome 11p15.5 and Comparison to Human Dopaminergic Neurons
Source: PLoS One. 2008 Jan 9;3(1):e1422. doi: 10.1371/journal.pone.0001422 (PMC2170519; doi:10.1371/journal.pone.0001422)
Supplement: Table S3 — Genes uniquely expressed in NCAM+ cells (0.43 MB DOC) [file pone.0001422.s003.doc]

**Table S3. Genes uniquely expressed in NCAM^+^ cells**

| **Category** | **Accession** | **Gene name** | **Symbol** | **NCAM^+^** | **BG02** | **Pooled hESCs** |
| --- | --- | --- | --- | --- | --- | --- |
| ECM and TM | Hs.443625 | Collagen, type III, alpha 1 (Ehlers-Danlos syndrome type IV, autosomal dominant) | COL3A1 | 15001 | 215 | 346 |
| ECM and TM | Hs.156316 | Decorin | DCN | 4514 | 0 | 16 |
| ECM and TM | Hs.156316 | Decorin | DCN | 4514 | 0 | 16 |
| ECM and TM | Hs.76152 | Aquaporin 1 (channel-forming integral protein, 28kDa) | AQP1 | 3523 | 7 | 0 |
| ECM and TM | Hs.24601 | Fibulin 1 | FBLN1 | 3038 | 211 | 30 |
| ECM and TM | Hs.406475 | Lumican | LUM | 2798 | 18 | 15 |
| ECM and TM | Hs.473721 | Solute carrier family 2 (facilitated glucose transporter), member 1 | SLC2A1 | 2592 | 101 | 178 |
| ECM and TM | Hs.210283 | Collagen, type V, alpha 1 | COL5A1 | 1567 | 7 | 45 |
| ECM and TM | Hs.361323 | ATP-binding cassette, sub-family F (GCN20), member 3 | ABCF3 | 1165 | 72 | 29 |
| ECM and TM | Hs.521731 | Angiopoietin-like 2 | ANGPTL2 | 717 | 8 | 0 |
| ECM and TM | Hs.381870 | EGF-containing fibulin-like extracellular matrix protein 2 | EFEMP2 | 676 | 20 | 31 |
| ECM and TM | Hs.532685 | Carboxypeptidase X (M14 family) | CPXM | 585 | 8 | 33 |
| ECM and TM | Hs.471751 | Chemokine orphan receptor 1 | CMKOR1 | 541 | 0 | 0 |
| ECM and TM | Hs.514535 | Lectin, galactoside-binding, soluble, 3 binding protein | LGALS3BP | 529 | 0 | 19 |
| ECM and TM | Hs.529285 | Solute carrier family 40 (iron-regulated transporter), member 1 | SLC40A1 | 498 | 0 | 13 |
| ECM and TM | Hs.464422 | Collectin sub-family member 12 | COLEC12 | 466 | 17 | 17 |
| ECM and TM | Hs.164226 | Thrombospondin 1 | THBS1 | 421 | 32 | 2 |
| ECM and TM | Hs.351874 | Major histocompatibility complex, class II, DO alpha | HLA-DOA | 415 | 23 | 0 |
| ECM and TM | Hs.532768 | Serpin peptidase inhibitor, clade F (alpha-2 antiplasmin, pigment epithelium derived factor), member 1 | SERPINF1 | 414 | 8 | 7 |
| ECM and TM | Hs.482390 | Transforming growth factor, beta receptor III (betaglycan, 300kDa) | TGFBR3 | 371 | 0 | 0 |
| ECM and TM | Hs.3416 | Adipose differentiation-related protein | ADFP | 358 | 34 | 16 |
| ECM and TM | Hs.471751 | Chemokine orphan receptor 1 | CMKOR1 | 541 | 0 | 0 |
| ECM and TM | Hs.499941 | Tetraspanin 15 | TM4SF15 | 355 | 3 | 0 |
| ECM and TM | Hs.195727 | CD248 antigen, endosialin | CD248 | 336 | 0 | 0 |
| ECM and TM | Hs.365706 | Matrix Gla protein | MGP | 316 | 0 | 6 |
| ECM and TM | Hs.369840 | Nidogen 2 (osteonidogen) | NID2 | 315 | 0 | 0 |
| ECM and TM | Hs.9999 | Epithelial membrane protein 3 | EMP3 | 283 | 0 | 0 |
| ECM and TM | Hs.446438 | G protein-coupled receptor, family C, group 5, member C | GPRC5C | 282 | 0 | 0 |
| ECM and TM | Hs.513147 | Solute carrier family 7 (cationic amino acid transporter, y+ system), member 7 | SLC7A7 | 264 | 0 | 11 |
| ECM and TM | Hs.533136 | Low density lipoprotein receptor-related protein associated protein 1 | LRPAP1 | 255 | 0 | 0 |
| ECM and TM | Hs.516578 | Tissue factor pathway inhibitor (lipoprotein-associated coagulation inhibitor) | TFPI | 237 | 0 | 0 |
| ECM and TM | Hs.171057 | Immunoglobulin superfamily, member 3 | IGSF3 | 222 | 0 | 7 |
| ECM and TM | Hs.513779 | Cysteine-rich secretory protein LCCL domain containing 2 | DKFZP434B044 | 216 | 0 | 0 |
| ECM and TM | Hs.396783 | Solute carrier family 9 (sodium/hydrogen exchanger), member 3 regulator 1 | SLC9A3R1 | 210 | 0 | 0 |
| ECM and TM | Hs.504031 | FXYD domain containing ion transport regulator 6 | FXYD6 | 203 | 11 | 3 |
| ECM and TM | Hs.302963 | Spondin 2, extracellular matrix protein | SPON2 | 200 | 0 | 4 |
| ECM and TM | Hs.403790 | Solute carrier family 25, member 28 | SLC25A28 | 200 | 0 | 2 |
| ECM and TM | Hs.521934 | Solute carrier family 39 (zinc transporter), member 4 | SLC39A4 | 183 | 0 | 0 |
| ECM and TM | Hs.500761 | Solute carrier family 16 (monocarboxylic acid transporters), member 3 | SLC16A3 | 176 | 0 | 0 |
| ECM and TM | Hs.166486 | Chloride channel 5 (nephrolithiasis 2, X-linked, Dent disease) | CLCN5 | 163 | 0 | 5 |
| ECM and TM | Hs.518921 | Nephronectin | NPNT | 157 | 0 | 0 |
| ECM and TM | Hs.151641 | Leucine rich repeat containing 32 | GARP | 144 | 0 | 1 |
| ECM and TM | Hs.353001 | Collagen, type VIII, alpha 2 | COL8A2 | 144 | 0 | 0 |
| ECM and TM | Hs.500572 | Fer-1-like 3, myoferlin (C. elegans) | FER1L3 | 134 | 0 | 2 |
| ECM and TM | Hs.158954 | CD300 antigen like family member E | CD300LE | 108 | 0 | 0 |
| ECM and TM | Hs.253994 | FRAS1 related extracellular matrix protein 2 | FREM2 | 108 | 3 | 5 |
| ECM and TM | Hs.252189 | Syndecan 4 (amphiglycan, ryudocan) | SDC4 | 107 | 0 | 0 |
| ECM and TM | Hs.188859 | G protein-coupled receptor 20 | GPR20 | 104 | 0 | 0 |
| metabolic | Hs.370689 | UPF2 regulator of nonsense transcripts homolog (yeast) | UPF2 | 3333 | 21 | 19 |
| metabolic | Hs.302145 | Hemoglobin, gamma G | HBG2 | 1192 | 23 | 105 |
| metabolic | Hs.195080 | Endothelin converting enzyme 1 | ECE1 | 935 | 31 | 81 |
| metabolic | Hs.418123 | Cathepsin L-like 3 | CTSL | 614 | 0 | 35 |
| metabolic | Hs.444709 | Arylsulfatase I | ARSI | 412 | 0 | 0 |
| metabolic | Hs.34421 | UDP-N-acetyl-alpha-D-galactosamine:polypeptide N-acetylgalactosaminyltransferase 10 (GalNAc-T10) | GALNT10 | 339 | 0 | 25 |
| metabolic | Hs.6126 | Mannosidase, beta A, lysosomal-like | MANBAL | 302 | 0 | 14 |
| metabolic | Hs.439463 | AE binding protein 1 | AEBP1 | 270 | 3 | 8 |
| metabolic | Hs.437388 | Phosphatidylinositol glycan, class T | PIGT | 241 | 0 | 8 |
| metabolic | Hs.54941 | Phosphorylase kinase, alpha 2 (liver) | PHKA2 | 239 | 0 | 6 |
| metabolic | Hs.117545 | Phosphodiesterase 4D, cAMP-specific (phosphodiesterase E3 dunce homolog, Drosophila) | PDE4D | 210 | 0 | 0 |
| metabolic | Hs.421194 | Tyrosylprotein sulfotransferase 1 | TPST1 | 193 | 0 | 3 |
| metabolic | Hs.95120 | Cytoglobin | CYGB | 188 | 0 | 0 |
| metabolic | Hs.511767 | Indolethylamine N-methyltransferase | INMT | 183 | 0 | 0 |
| metabolic | Hs.443728 | SH3 domain containing ring finger 2 | SH3RF2 | 181 | 0 | 0 |
| metabolic | Hs.471917 | Proteasome (prosome, macropain) inhibitor subunit 1 (PI31) | PSMF1 | 167 | 12 | 6 |
| metabolic | Hs.460587 | Sulfotransferase family, cytosolic, 1A, phenol-preferring, member 3 | SULT1A3 | 160 | 0 | 0 |
| metabolic | Hs.109 | Dipeptidase 1 (renal) | DPEP1 | 152 | 14 | 0 |
| metabolic | Hs.374446 | Rho GTPase activating protein 23 | ARHGAP23 | 152 | 0 | 0 |
| metabolic | Hs.425801 | Dual specificity phosphatase 23 | DUSP23 | 152 | 0 | 0 |
| metabolic | Hs.400659 | Pseudouridylate synthase-like 1 | PUSL1 | 147 | 0 | 0 |
| metabolic | Hs.78068 | Carboxypeptidase Z | CPZ | 144 | 0 | 0 |
| metabolic | Hs.425023 | Serine palmitoyltransferase, long chain base subunit 2-like (aminotransferase 2) | SPTLC2L | 140 | 12 | 0 |
| metabolic | Hs.288284 | PQ loop repeat containing 1 | PQLC1 | 135 | 0 | 0 |
| metabolic | Hs.444709 | Arylsulfatase I | ARSI | 412 | 0 | 0 |
| metabolic | Hs.491336 | Elongation protein 3 homolog (S. cerevisiae) | ELP3 | 130 | 0 | 1 |
| metabolic | Hs.409911 | Tafazzin (cardiomyopathy, dilated 3A (X-linked); endocardial fibroelastosis 2; Barth syndrome) | TAZ | 122 | 9 | 0 |
| metabolic | Hs.279837 | Glutathione S-transferase M2 (muscle) | GSTM2 | 114 | 0 | 0 |
| metabolic | Hs.516370 | Carbohydrate sulfotransferase 10 | CHST10 | 114 | 0 | 0 |
| metabolic | Hs.195471 | 6-phosphofructo-2-kinase/fructose-2,6-biphosphatase 3 | PFKFB3 | 111 | 0 | 1 |
| metabolic | Hs.512810 | Diacylglycerol O-acyltransferase homolog 1 (mouse) | DGAT1 | 111 | 0 | 10 |
| metabolic | Hs.100915 | Peroxisomal biogenesis factor 16 | PEX16 | 104 | 0 | 0 |
| metabolic | Hs.31074 | N-sulfoglucosamine sulfohydrolase (sulfamidase) | SGSH | 104 | 0 | 0 |
| metabolic | Hs.98381 | Protease, serine, 35 | PRSS35 | 101 | 8 | 0 |
| mitochondria | Hs.501794 | ATPase type 13A1 | ATP13A1 | 203 | 0 | 8 |
| nuclear | Hs.152531 | Heart and neural crest derivatives expressed 1 | HAND1 | 6522 | 0 | 0 |
| nuclear | Hs.84136 | Paired-like homeodomain transcription factor 1 | PITX1 | 2018 | 5 | 2 |
| nuclear | Hs.2780 | Jun D proto-oncogene | JUND | 1432 | 35 | 87 |
| nuclear | Hs.424414 | Msh homeo box homolog 1 (Drosophila) | MSX1 | 1098 | 0 | 13 |
| nuclear | Hs.405961 | CAMP responsive element binding protein 3-like 1 | CREB3L1 | 1062 | 32 | 9 |
| nuclear | Hs.25647 | V-fos FBJ murine osteosarcoma viral oncogene homolog | FOS | 941 | 26 | 14 |
| nuclear | Hs.92282 | Paired-like homeodomain transcription factor 2 | PITX2 | 585 | 27 | 28 |
| nuclear | Hs.530461 | Histone 2, H2aa | HIST2H2AA | 543 | 0 | 17 |
| nuclear | Hs.226117 | H1 histone family, member 0 | H1F0 | 390 | 28 | 8 |
| nuclear | Hs.466693 | Sirtuin (silent mating type information regulation 2 homolog) 2 (S. cerevisiae) | SIRT2 | 353 | 0 | 0 |
| nuclear | Hs.519162 | BTG family, member 2 | BTG | 337 | 0 | 20 |
| nuclear | Hs.360174 | Snail homolog 2 (Drosophila) | SNAI2 | 332 | 0 | 0 |
| nuclear | Hs.80409 | Growth arrest and DNA-damage-inducible, alpha | GADD45A | 313 | 0 | 0 |
| nuclear | Hs.134859 | V-maf musculoaponeurotic fibrosarcoma oncogene homolog (avian) | MAF | 296 | 0 | 19 |
| nuclear | Hs.444468 | CTD (carboxy-terminal domain, RNA polymerase II, polypeptide A) small phosphatase 1 | CTDSP1 | 282 | 0 | 0 |
| nuclear | Hs.50130 | Necdin homolog (mouse) | NDN | 270 | 23 | 19 |
| nuclear | Hs.459153 | Basonuclin 1 | BNC1 | 263 | 0 | 0 |
| nuclear | Hs.220971 | FOS-like antigen 2 | FOSL2 | 251 | 8 | 7 |
| nuclear | Hs.502875 | V-rel reticuloendotheliosis viral oncogene homolog A, nuclear factor of kappa light polypeptide gene enhancer in B-cells 3, p65 (avian) | RELA | 225 | 7 | 21 |
| nuclear | Hs.532669 | Homeo box B4 | HOXB4 | 212 | 0 | 0 |
| nuclear | Hs.532755 | Gene trap locus 3 (mouse) | GTL3 | 209 | 0 | 0 |
| nuclear | Hs.269898 | SERTA domain containing 1 | SERTAD1 | 206 | 0 | 1 |
| nuclear | Hs.108112 | Polymerase (DNA directed), epsilon 3 (p17 subunit) | POLE3 | 193 | 4 | 4 |
| nuclear | Hs.419259 | REC8-like 1 (yeast) | REC8L1 | 193 | 0 | 7 |
| nuclear | Hs.517970 | Inositol hexaphosphate kinase 1 | IHPK1 | 189 | 0 | 0 |
| nuclear | Hs.326035 | Early growth response 1 | EGR1 | 182 | 0 | 0 |
| nuclear | Hs.48950 | Dapper, antagonist of beta-catenin, homolog 1 (Xenopus laevis) | DACT1 | 182 | 2 | 2 |
| nuclear | Hs.515412 | SERTA domain containing 3 | SERTAD3 | 159 | 0 | 0 |
| nuclear | Hs.129895 | T-box 3 (ulnar mammary syndrome) | TBX3 | 154 | 0 | 0 |
| nuclear | Hs.5710 | Cellular repressor of E1A-stimulated genes 1 | CREG1 | 146 | 7 | 6 |
| nuclear | Hs.514746 | GATA binding protein 6 | GATA6 | 142 | 11 | 11 |
| nuclear | Hs.388245 | Heart and neural crest derivatives expressed 2 | HAND2 | 141 | 0 | 0 |
| nuclear | Hs.12107 | Chromatin modifying protein 2A | BC-2 | 138 | 0 | 0 |
| nuclear | Hs.153629 | MADS box transcription enhancer factor 2, polypeptide B (myocyte enhancer factor 2B) | MEF2B | 129 | 0 | 4 |
| nuclear | Hs.46700 | Inhibitor of growth family, member 1 | ING1 | 119 | 0 | 0 |
| nuclear | Hs.523230 | Polymerase (DNA directed), lambda | POLL | 108 | 0 | 10 |
| nuclear | Hs.469280 | AT hook, DNA binding motif, containing 1 | DJ159A19.3 | 102 | 0 | 3 |
| Ribosomal | Hs.8562 | Ribonuclease P 25kDa subunit | RPP25 | 539 | 0 | 0 |
| Ribosomal | Hs.412370 | Ribosomal protein L9 | RPL9 | 407 | 0 | 0 |
| signal | Hs.523414 | Insulin-like growth factor 2 (somatomedin A) | IGF2 | 56981 | 8 | 304 |
| signal | Hs.111779 | Secreted protein, acidic, cysteine-rich (osteonectin) | SPARC | 5467 | 335 | 79 |
| signal | Hs.106070 | Cyclin-dependent kinase inhibitor 1C (p57, Kip2) | CDKN1C | 2194 | 7 | 147 |
| signal | Hs.118110 | Bone marrow stromal cell antigen 2 | BST2 | 1414 | 35 | 56 |
| signal | Hs.128453 | Frizzled-related protein | FRZB | 1344 | 0 | 43 |
| signal | Hs.380906 | Myeloid-associated differentiation marker | MYADM | 1031 | 41 | 6 |
| signal | Hs.502876 | Ras homolog gene family, member B | RHOB | 918 | 0 | 5 |
| signal | Hs.334629 | Sarcolipin | SLN | 754 | 0 | 0 |
| signal | Hs.279832 | Docking protein 4 | DOK4 | 652 | 11 | 0 |
| signal | Hs.463110 | Annexin A8 | ANXA8 | 592 | 0 | 0 |
| signal | Hs.110571 | Growth arrest and DNA-damage-inducible, beta | GADD45B | 494 | 18 | 0 |
| signal | Hs.370950 | AXIN1 up-regulated 1 | AXUD1 | 490 | 11 | 0 |
| signal | Hs.533717 | Delta-like 1 homolog (Drosophila) | DLK1 | 476 | 0 | 0 |
| signal | Hs.3989 | Plexin B2 | PLXNB2 | 463 | 0 | 9 |
| signal | Hs.171695 | Dual specificity phosphatase 1 | DUSP1 | 376 | 0 | 24 |
| signal | Hs.549043 | Insulin-like growth factor 2 (somatomedin A) | IGF2 | 345 | 0 | 0 |
| signal | Hs.1274 | Bone morphogenetic protein 1 | BMP1 | 331 | 11 | 3 |
| signal | Hs.112631 | Slit homolog 3 (Drosophila) | SLIT3 | 329 | 3 | 20 |
| signal | Hs.549184 | ADAM metallopeptidase with thrombospondin type 1 motif, 9 | ADAMTS9 | 324 | 12 | 22 |
| signal | Hs.444947 | Tribbles homolog 1 (Drosophila) | TRIB1 | 316 | 0 | 5 |
| signal | Hs.534315 | Guanine nucleotide binding protein (G protein), beta polypeptide 3 | GNB3 | 308 | 0 | 0 |
| signal | Hs.861 | Mitogen-activated protein kinase 3 | MAPK3 | 305 | 27 | 0 |
| signal | Hs.480938 | LPS-responsive vesicle trafficking, beach and anchor containing | LRBA | 293 | 12 | 14 |
| signal | Hs.146585 | O-acyltransferase (membrane bound) domain containing 4 | LEPROTL1 | 288 | 20 | 6 |
| signal | Hs.26312 | Leucine rich repeat neuronal 5 | LRRN5 | 285 | 5 | 0 |
| signal | Hs.29802 | Slit homolog 2 (Drosophila) | SLIT2 | 281 | 0 | 0 |
| signal | Hs.102336 | Rho GTPase activating protein 8 | ARHGAP8 | 279 | 3 | 19 |
| signal | Hs.303116 | Stromal cell-derived factor 2-like 1 | SDF2L1 | 259 | 12 | 23 |
| signal | Hs.465498 | Thioredoxin-like 4A | TXNL4A | 237 | 0 | 0 |
| signal | Hs.475353 | LIM and cysteine-rich domains 1 | LMCD1 | 233 | 0 | 0 |
| signal | Hs.109437 | Hormonally upregulated Neu-associated kinase | HUNK | 232 | 16 | 2 |
| signal | Hs.12923 | Signal peptide, CUB domain, EGF-like 3 | SCUBE3 | 231 | 0 | 1 |
| signal | Hs.252820 | Placental growth factor, vascular endothelial growth factor-related protein | PGF | 231 | 0 | 18 |
| signal | Hs.494457 | Ninjurin 1 | NINJ1 | 226 | 0 | 0 |
| signal | Hs.18676 | Sprouty homolog 2 (Drosophila) | SPRY2 | 224 | 7 | 13 |
| signal | Hs.521651 | Stathmin-like 2 | STMN2 | 220 | 2 | 0 |
| signal | Hs.494875 | Regulator of G-protein signalling 3 | RGS3 | 191 | 0 | 0 |
| signal | Hs.523309 | BCL2-associated athanogene 3 | BAG3 | 186 | 8 | 6 |
| signal | Hs.20315 | Interferon-induced protein with tetratricopeptide repeats 1 | IFIT1 | 184 | 0 | 2 |
| signal | Hs.523045 | Calcium/calmodulin-dependent protein kinase (CaM kinase) II gamma | CAMK2G | 184 | 2 | 7 |
| signal | Hs.133421 | Leukemia inhibitory factor receptor | LIFR | 182 | 0 | 8 |
| signal | Hs.533613 | Troponin T type 2 (cardiac) | TNNT2 | 181 | 3 | 0 |
| signal | Hs.320890 | Troponin I type 1 (skeletal, slow) | TNNI1 | 172 | 0 | 0 |
| signal | Hs.68879 | Bone morphogenetic protein 4 | BMP4 | 161 | 0 | 2 |
| signal | Hs.1832 | Neuropeptide Y | NPY | 160 | 0 | 0 |
| signal | Hs.546410 | Cripto, FRL-1, cryptic family 1 | CFC1 | 160 | 0 | 0 |
| signal | Hs.407909 | SH3-domain binding protein 1 | SH3BP1 | 152 | 0 | 1 |
| signal | Hs.1027 | Ras-related associated with diabetes | RRAD | 144 | 0 | 0 |
| signal | Hs.483444 | Chemokine (C-X-C motif) ligand 14 | CXCL14 | 144 | 0 | 0 |
| signal | Hs.502659 | Ras homolog gene family, member C | RHOC | 141 | 0 | 0 |
| signal | Hs.270055 | SH3-domain GRB2-like 3 | SH3GL3 | 137 | 0 | 2 |
| signal | Hs.98259 | Sterile alpha motif domain containing 4 | SAMD4 | 135 | 4 | 5 |
| signal | Hs.514477 | Lethal giant larvae homolog 2 (Drosophila) | LLGL2 | 131 | 0 | 0 |
| signal | Hs.523424 | Tumor suppressing subtransferable candidate 4 | TSSC4 | 128 | 0 | 8 |
| signal | Hs.495704 | RAB9A, member RAS oncogene family | RAB9A | 120 | 7 | 0 |
| signal | Hs.184482 | Low density lipoprotein receptor adaptor protein 1 | ARH | 118 | 0 | 2 |
| signal | Hs.89983 | Mannan-binding lectin serine peptidase 1 (C4/C2 activating component of Ra-reactive factor) | MASP1 | 118 | 0 | 0 |
| signal | Hs.81256 | S100 calcium binding protein A4 (calcium protein, calvasculin, metastasin, murine placental homolog) | S100A4 | 116 | 0 | 0 |
| signal | Hs.149168 | Tumor necrosis factor receptor superfamily, member 19 | TNFRSF19 | 113 | 0 | 0 |
| signal | Hs.351413 | RAB37, member RAS oncogene family | RAB37 | 111 | 0 | 0 |
| signal | Hs.432453 | Mitogen-activated protein kinase kinase kinase 8 | MAP3K8 | 110 | 0 | 7 |
| signal | Hs.546538 | Crumbs homolog 2 (Drosophila) | CRB2 | 108 | 0 | 0 |
| signal | Hs.77422 | Proteolipid protein 2 (colonic epithelium-enriched) | PLP2 | 107 | 3 | 5 |
| signal | Hs.355888 | Phospholipase C, beta 2 | PLCB2 | 104 | 0 | 2 |
| signal | Hs.435001 | Kruppel-like factor 10 | KLF10 | 104 | 0 | 2 |
| structual | Hs.514167 | Keratin 19 | KRT19 | 1504 | 143 | 6 |
| structual | Hs.296049 | Microfibrillar-associated protein 4 | MFAP4 | 879 | 7 | 1 |
| structual | Hs.463300 | Myosin, light polypeptide 4, alkali; atrial, embryonic | MYL4 | 874 | 36 | 0 |
| structual | Hs.58414 | Filamin C, gamma (actin binding protein 280) | FLNC | 733 | 19 | 8 |
| structual | Hs.474797 | CDC42 effector protein (Rho GTPase binding) 1 | CDC42EP1 | 350 | 0 | 0 |
| structual | Hs.511768 | Dynactin 3 (p22) | DCTN3 | 317 | 0 | 0 |
| structual | Hs.98791 | ARP1 actin-related protein 1 homolog B, centractin beta (yeast) | ACTR1B | 313 | 16 | 0 |
| structual | Hs.511686 | Talin 2 | TLN2 | 242 | 5 | 19 |
| structual | Hs.3903 | CDC42 effector protein (Rho GTPase binding) 4 | CDC42EP4 | 239 | 0 | 19 |
| structual | Hs.64746 | Chloride intracellular channel 3 | CLIC3 | 232 | 0 | 0 |
| unknown | Hs.551588 | Transcribed locus (syntaxin 7) |  | 63838 | 0 | 4 |
| unknown | Hs.19193 | Hypothetical gene supported by BC009385 |  | 997 | 0 | 9 |
| unknown | Hs.473109 | Chromosome 11 open reading frame 9 | C11orf9 | 816 | 32 | 0 |
| unknown | Hs.109798 | Chromosome 6 open reading frame 48 | C6orf48 | 747 | 0 | 62 |
| unknown | Hs.288232 | FLJ22642 fis, clone HSI06970 |  | 554 | 35 | 29 |
| unknown | Hs.4055 | Kruppel-like factor 6 | KLF6 | 527 | 28 | 43 |
| unknown | Hs.534052 | Zinc finger protein 36, C3H type, homolog (mouse) | ZFP36 | 439 | 20 | 0 |
| unknown | Hs.446315 | Chromosome 10 open reading frame 45 | C10orf45 | 431 | 0 | 28 |
| unknown | Hs.182626 | Chromosome 22 open reading frame 5 | C22orf5 | 324 | 23 | 20 |
| unknown | Hs.435535 | Zinc finger protein 395 | ZNF395 | 234 | 19 | 23 |
| unknown | Hs.509637 | Pleckstrin homology domain containing, family G (with RhoGef domain) member 3 | PLEKHG3 | 217 | 14 | 0 |
| unknown | Hs.424711 | Hypothetical protein FLJ22313 | FLJ22313 | 183 | 0 | 11 |
| unknown | Hs.103315 | Zinc finger protein 384 | ZNF384 | 179 | 0 | 14 |
| unknown | Hs.211282 | Cysteine-rich with EGF-like domains 2 | MGC11256 | 174 | 0 | 2 |
| unknown | Hs.479491 | Chromosome 1 open reading frame 139 | FLJ23091 | 172 | 0 | 8 |
| unknown | Hs.534843 | Hypothetical LOC402573 | MGC43036 | 166 | 0 | 0 |
| unknown | Hs.381134 | Similar to RIKEN cDNA 1810059G22 | LOC374395 | 161 | 5 | 0 |
| unknown | Hs.471918 | Zinc finger CCCH-type containing 12A | ZC3H12A | 158 | 2 | 0 |
| unknown | Hs.323213 | Yip1 domain family, member 2 | YIPF2 | 155 | 0 | 0 |
| unknown | Hs.509923 | Chromosome 14 open reading frame 43 | C14orf43 | 151 | 14 | 0 |
| unknown | Hs.363138 | Solute carrier family 27 (fatty acid transporter), member 1 | SLC27A1 | 150 | 0 | 0 |
| unknown | Hs.513491 | Yippee-like 3 (Drosophila) | YPEL3 | 150 | 0 | 1 |
| unknown | Hs.30579 | LOC441207 | LOC441207 | 144 | 0 | 0 |
| unknown | Hs.470457 | Transcribed locus |  | 140 | 0 | 2 |
| unknown | Hs.522334 | Sushi, von Willebrand factor type A, EGF and pentraxin domain containing 1 | C9orf13 | 138 | 0 | 8 |
| unknown | Hs.469154 | WD and tetratricopeptide repeats 1 | WDTC1 | 137 | 0 | 3 |
| unknown | Hs.283398 | Ankyrin repeat domain 38 | ANKRD38 | 132 | 0 | 0 |
| unknown | Hs.435080 | Family with sequence similarity 43, member A | FAM43A | 132 | 0 | 0 |
| unknown | Hs.16157 | Chromosome 14 open reading frame 133 | C14orf133 | 131 | 0 | 3 |
| unknown | Hs.444950 | TBC1 domain family, member 10A | TBC1D10 | 131 | 7 | 3 |
| unknown | Hs.251699 | Similar to expressed sequence AI836003 |  | 123 | 0 | 0 |
| unknown | Hs.272848 | Hypothetical protein FLJ21019 | FLJ21019 | 118 | 0 | 2 |
| unknown | Hs.34333 | Collagen and calcium binding EGF domains 1 | CCBE1 | 114 | 3 | 0 |
| unknown | Hs.497417 | KIAA0317 | KIAA0317 | 114 | 0 | 11 |
| unknown | Hs.417157 | Hypothetical protein MGC14376 | MGC14376 | 109 | 0 | 0 |
| unknown | Hs.298227 | Chromosome 14 open reading frame 174 | C14orf174 | 108 | 0 | 0 |
| unknown | Hs.369613 | Chromosome 19 open reading frame 20 | C19orf20 | 102 | 0 | 0 |
