# Supplementary material for: Gene Expression Profile of Neuronal Progenitor Cells Derived from hESCs: Activation of Chromosome 11p15.5 and Comparison to Human Dopaminergic Neurons
Source: PLoS One. 2008 Jan 9;3(1):e1422. doi: 10.1371/journal.pone.0001422 (PMC2170519; doi:10.1371/journal.pone.0001422)
Supplement: Table S4 — Cytogentic map locations for highly-expressed genes in PSA-NCAM+ FACS-sorted cells (0.29 MB DOC) [file pone.0001422.s004.doc]

**Table S4.** **Cytogentic map locations for highly-expressed genes in PSA-NCAM^+^ FACS-sorted cells**

| **Gene name** | **Symbol** | **CytoGenetic Map** | **Chromosome** |
| --- | --- | --- | --- |
| Immunoglobulin superfamily, member 3 | IGSF3 | 1p13 | 1 |
| Ras homolog gene family, member C | RHOC | 1p13.1 | 1 |
| Glutathione S-transferase M2 (muscle) | GSTM2 | 1p13.3 | 1 |
| Growth arrest and DNA-damage-inducible, alpha | GADD45A | 1p31.2-p31.1 | 1 |
| Ankyrin repeat domain 38 | ANKRD38 | 1p31.3 | 1 |
| Chromosome 1 open reading frame 139 | GPR177 | 1p31.3 | 1 |
| Transforming growth factor, beta receptor III (betaglycan, 300kDa) | TGFBR3 | 1p33-p32 | 1 |
| Collagen, type VIII, alpha 2 | COL8A2 | 1p34.2 | 1 |
| Zinc finger CCCH-type containing 12A | ZC3H12A | 1p34.3 | 1 |
| Solute carrier family 2 (facilitated glucose transporter), member 1 | SLC2A1 | 1p35-p31.3 | 1 |
| Endothelin converting enzyme 1 | ECE1 | 1p36.1 | 1 |
| WD and tetratricopeptide repeats 1 | WDTC1 | 1p36.11 | 1 |
| AT hook, DNA binding motif, containing 1 | AHDC1 | 1p36.13 | 1 |
| Pseudouridylate synthase-like 1 | PUSL1 | 1p36.33 | 1 |
| Low density lipoprotein receptor adaptor protein 1 | LDLRAP1 | 1p36-p35 | 1 |
| S100 calcium binding protein A4 (calcium protein, calvasculin, metastasin, murine placental homolog) | S100A4 | 1q21 | 1 |
| Histone 2, H2aa | HIST2H2AA | 1q21.2 | 1 |
| Dual specificity phosphatase 23 | DUSP23 | 1q23.2 | 1 |
| Cellular repressor of E1A-stimulated genes 1 | CREG1 | 1q24 | 1 |
| Troponin I type 1 (skeletal, slow) | TNNI1 | 1q31.3 | 1 |
| BTG family, member 2 | BTG2 | 1q32 | 1 |
| Troponin T type 2 (cardiac) | TNNT2 | 1q32 | 1 |
| Leucine rich repeat neuronal 5 | LRRN5 | 1q32.1 | 1 |
| FOS-like antigen 2 | FOSL2 | 2p23.3 | 2 |
| Ras homolog gene family, member B | RHOB | 2p24 | 2 |
| ARP1 actin-related protein 1 homolog B, centractin beta (yeast) | ACTR1B | 2q11.1-q11.2 | 2 |
| Carbohydrate sulfotransferase 10 | CHST10 | 2q11.2 | 2 |
| Cripto, FRL-1, cryptic family 1 | CFC1 | 2q21.1 | 2 |
| COBL-like 1 | COBLL1 | 2q24.3 | 2 |
| Collagen, type III, alpha 1 (Ehlers-Danlos syndrome type IV, autosomal dominant) | COL3A1 | 2q31 | 2 |
| Tissue factor pathway inhibitor (lipoprotein-associated coagulation inhibitor) | TFPI | 2q31-q32.1 | 2 |
| Solute carrier family 40 (iron-regulated transporter), member 1 | SLC40A1 | 2q32 | 2 |
| CTD (carboxy-terminal domain, RNA polymerase II, polypeptide A) small phosphatase 1 | CTDSP1 | 2q35 | 2 |
| Chemokine orphan receptor 1 | CMKOR1 | 2q37.3 | 2 |
| Frizzled-related protein | FRZB | 2qter | 2 |
| ADAM metallopeptidase with thrombospondin type 1 motif, 9 | ADAMTS9 | 3p14.3-p14.2 | 3 |
| Inositol hexaphosphate kinase 1 | IHPK1 | 3p21.31 | 3 |
| AXIN1 up-regulated 1 | AXUD1 | 3p22 | 3 |
| LIM and cysteine-rich domains 1 | LMCD1 | 3p26-p24 | 3 |
| ATP-binding cassette, sub-family F (GCN20), member 3 | ABCF3 | 3q27.1 | 3 |
| Mannan-binding lectin serine peptidase 1 (C4/C2 activating component of Ra-reactive factor) | MASP1 | 3q27-q28 | 3 |
| Family with sequence similarity 43, member A | FAM43A | 3q29 | 3 |
| Ribosomal protein L9 | RPL9 | 4p13 | 4 |
| Slit homolog 2 (Drosophila) | SLIT2 | 4p15.2 | 4 |
| Carboxypeptidase Z | CPZ | 4p16.1 | 4 |
| Low density lipoprotein receptor-related protein associated protein 1 | LRPAP1 | 4p16.3 | 4 |
| Spondin 2, extracellular matrix protein | SPON2 | 4p16.3 | 4 |
| Msh homeo box homolog 1 (Drosophila) | MSX1 | 4p16.3-p16.1 | 4 |
| Nephronectin | NPNT | 4q24 | 4 |
| Paired-like homeodomain transcription factor 2 | PITX2 | 4q25-q27 | 4 |
| LPS-responsive vesicle trafficking, beach and anchor containing | LRBA | 4q31.3 | 4 |
| Heart and neural crest derivatives expressed 2 | HAND2 | 4q33 | 4 |
| Leukemia inhibitory factor receptor | LIFR | 5p13-p12 | 5 |
| Phosphodiesterase 4D, cAMP-specific (phosphodiesterase E3 dunce homolog, Drosophila) | PDE4D | 5q12 | 5 |
| Serine incorporator 5 | SERINC5 | 5q14.1 | 5 |
| Paired-like homeodomain transcription factor 1 | PITX1 | 5q31 | 5 |
| Chemokine (C-X-C motif) ligand 14 | CXCL14 | 5q31 | 5 |
| Early growth response 1 | EGR1 | 5q31.1 | 5 |
| Secreted protein, acidic, cysteine-rich (osteonectin) | SPARC | 5q31.3-q32 | 5 |
| SH3 domain containing ring finger 2 | SH3RF2 | 5q32 | 5 |
| Heart and neural crest derivatives expressed 1 | HAND1 | 5q33 | 5 |
| Arylsulfatase I | ARSI | 5q33.1 | 5 |
| UDP-N-acetyl-alpha-D-galactosamine:polypeptide N-acetylgalactosaminyltransferase 10 (GalNAc-T10) | GALNT10 | 5q33.2 | 5 |
| Dual specificity phosphatase 1 | DUSP1 | 5q34 | 5 |
| Slit homolog 3 (Drosophila) | SLIT3 | 5q35 | 5 |
| Tumor necrosis factor receptor superfamily, member 21 | TNFRSF21 | 6p21.1-12.2 | 6 |
| Chromosome 6 open reading frame 48 | C6orf48 | 6p21.3 | 6 |
| Major histocompatibility complex, class II, DO alpha | HLA-DOA | 6p21.3 | 6 |
| Signal peptide, CUB domain, EGF-like 3 | SCUBE3 | 6p21.3 | 6 |
| Protease, serine, 35 | PRSS35 | 6q14.2 | 6 |
| LOC441207 | LOC441207 | 7 | 7 |
| AE binding protein 1 | AEBP1 | 7p13 | 7 |
| Aquaporin 1 (channel-forming integral protein, 28kDa) | AQP1 | 7p14 | 7 |
| HERPUD family member 2 | HERPUD2 | 7p14.2 | 7 |
| Neuropeptide Y | NPY | 7p15.1 | 7 |
| Indolethylamine N-methyltransferase | INMT | 7p15.3-p15.2 | 7 |
| TSC22 domain family, member 4 | TSC22D4 | 7p21-p15 | 7 |
| Tyrosylprotein sulfotransferase 1 | TPST1 | 7q11.21 | 7 |
| Filamin C, gamma (actin binding protein 280) | FLNC | 7q32-q35 | 7 |
| Bone morphogenetic protein 1 | BMP1 | 8p21 | 8 |
| Elongation protein 3 homolog (S. cerevisiae) | ELP3 | 8p21.1 | 8 |
| Zinc finger protein 395 | ZNF395 | 8p21.1 | 8 |
| O-acyltransferase (membrane bound) domain containing 4 | LEPROTL1 | 8p21.2-p21.1 | 8 |
| Snail homolog 2 (Drosophila) | SNAI2 | 8q11 | 8 |
| Stathmin-like 2 | STMN2 | 8q21.13 | 8 |
| Kruppel-like factor 10 | KLF10 | 8q22.2 | 8 |
| Tribbles homolog 1 (Drosophila) | TRIB1 | 8q24.13 | 8 |
| G protein-coupled receptor 20 | GPR20 | 8q24.2-q24.3 | 8 |
| Solute carrier family 39 (zinc transporter), member 4 | SLC39A4 | 8q24.3 | 8 |
| Diacylglycerol O-acyltransferase homolog 1 (mouse) | DGAT1 | 8q24.3 | 8 |
| Dynactin 3 (p22) | DCTN3 | 9p13 | 9 |
| Adipose differentiation-related protein | ADFP | 9p22.1 | 9 |
| Cathepsin L-like 3 | CTSL | 9q21-q22 | 9 |
| Ninjurin 1 | NINJ1 | 9q22 | 9 |
| Sushi, von Willebrand factor type A, EGF and pentraxin domain containing 1 | SVEP1 | 9q32 | 9 |
| Regulator of G-protein signalling 3 | RGS3 | 9q32 | 9 |
| Polymerase (DNA directed), epsilon 3 (p17 subunit) | POLE3 | 9q33 | 9 |
| Crumbs homolog 2 (Drosophila) | CRB2 | 9q33.2 | 9 |
| Angiopoietin-like 2 | ANGPTL2 | 9q34 | 9 |
| Collagen, type V, alpha 1 | COL5A1 | 9q34.2-q34.3 | 9 |
| Chloride intracellular channel 3 | CLIC3 | 9q34.3 | 9 |
| Mitogen-activated protein kinase kinase kinase 8 | MAP3K8 | 10p11.23 | 10 |
| Family with sequence similarity 107, member B | FAM107B | 10p13 | 10 |
| UPF2 regulator of nonsense transcripts homolog (yeast) | UPF2 | 10p14-p13 | 10 |
| 6-phosphofructo-2-kinase/fructose-2,6-biphosphatase 3 | PFKFB3 | 10p14-p15 | 10 |
| Kruppel-like factor 6 | KLF6 | 10p15 | 10 |
| Annexin A8 | ANXA8 | 10q11.2 | 10 |
| Tetraspanin 15 | TSPAN15 | 10q21.3 | 10 |
| Calcium/calmodulin-dependent protein kinase (CaM kinase) II gamma | CAMK2G | 10q22 | 10 |
| Polymerase (DNA directed), lambda | POLL | 10q23 | 10 |
| Solute carrier family 25, member 28 | SLC25A28 | 10q23-q24 | 10 |
| Fer-1-like 3, myoferlin (C. elegans) | FER1L3 | 10q24 | 10 |
| BCL2-associated athanogene 3 | BAG3 | 10q25.2-q26.2 | 10 |
| Interferon-induced protein with tetratricopeptide repeats 1 | IFIT1 | 10q25-q26 | 10 |
| CAMP responsive element binding protein 3-like 1 | CREB3L1 | 11p11.2 | 11 |
| Peroxisomal biogenesis factor 16 | PEX16 | 11p11.2 | 11 |
| Insulin-like growth factor 2 (somatomedin A) | IGF2 | 11p15.5 | 11 |
| Cyclin-dependent kinase inhibitor 1C (p57, Kip2) | CDKN1C | 11p15.5 | 11 |
| Hemoglobin, gamma G | HBG2 | 11p15.5 | 11 |
| Tumor suppressing subtransferable candidate 4 | TSSC4 | 11p15.5 | 11 |
| H19, imprinted maternally expressed untranslated mRNA | H19 | 11p15.5 | 11 |
| Similar to RIKEN cDNA 1810059G22 | LOC374395 | 11q12.3 | 11 |
| Chromosome 11 open reading frame 9 | C11orf9 | 11q12-q13.1 | 11 |
| CD248 antigen, endosialin | CD248 | 11q13 | 11 |
| EGF-containing fibulin-like extracellular matrix protein 2 | EFEMP2 | 11q13 | 11 |
| V-rel reticuloendotheliosis viral oncogene homolog A, nuclear factor of kappa light polypeptide gene enhancer in B-cells 3, p65 (avian) | RELA | 11q13 | 11 |
| Leucine rich repeat containing 32 | LRRC32 | 11q13.5-q14 | 11 |
| Sarcolipin | SLN | 11q22-q23 | 11 |
| FXYD domain containing ion transport regulator 6 | FXYD6 | 11q23.3 | 11 |
| Insulin-like growth factor 2 (somatomedin A) | IGF2 | 11p15.5 | 11 |
| Zinc finger protein 384 | ZNF384 | 12p12 | 12 |
| Triosephosphate isomerase 1 | TPI1 | 12p13 | 12 |
| Matrix Gla protein | MGP | 12p13.1-p12.3 | 12 |
| Similar to expressed sequence AI836003 | LOC387856 | 12q13.11 | 12 |
| Hypothetical gene supported by BC009385 | LOC400043 | 12q13.13 | 12 |
| Decorin | DCN | 12q21.33 | 12 |
| Lumican | LUM | 12q21.3-q22 | 12 |
| T-box 3 (ulnar mammary syndrome) | TBX3 | 12q24.1 | 12 |
| Tumor necrosis factor receptor superfamily, member 19 | TNFRSF19 | 13q12.11-q12.3 | 13 |
| FRAS1 related extracellular matrix protein 2 | FREM2 | 13q13.3 | 13 |
| Sprouty homolog 2 (Drosophila) | SPRY2 | 13q31.1 | 13 |
| Inhibitor of growth family, member 1 | ING1 | 13q34 | 13 |
| Solute carrier family 7 (cationic amino acid transporter, y+ system), member 7 | SLC7A7 | 14q11.2 | 14 |
| REC8-like 1 (yeast) | REC8L1 | 14q11.2-q12 | 14 |
| Nidogen 2 (osteonidogen) | NID2 | 14q21-q22 | 14 |
| Sterile alpha motif domain containing 4A | SAMD4A | 14q22.2 | 14 |
| Bone morphogenetic protein 4 | BMP4 | 14q22-q23 | 14 |
| Dapper, antagonist of beta-catenin, homolog 1 (Xenopus laevis) | DACT1 | 14q23.1 | 14 |
| Pleckstrin homology domain containing, family G (with RhoGef domain) member 3 | PLEKHG3 | 14q23.3 | 14 |
| Chromosome 14 open reading frame 174 | C14orf174 | 14q24.3 | 14 |
| Chromosome 14 open reading frame 43 | C14orf43 | 14q24.3 | 14 |
| V-fos FBJ murine osteosarcoma viral oncogene homolog | FOS | 14q24.3 | 14 |
| KIAA0317 | KIAA0317 | 14q24.3 | 14 |
| Chromosome 14 open reading frame 133 | C14orf133 | 14q24.3-q31 | 14 |
| Placental growth factor, vascular endothelial growth factor-related protein | PGF | 14q24-q31 | 14 |
| Delta-like 1 homolog (Drosophila) | DLK1 | 14q32 | 14 |
| Necdin homolog (mouse) | NDN | 15q11.2-q12 | 15 |
| Phospholipase C, beta 2 | PLCB2 | 15q15 | 15 |
| Thrombospondin 1 | THBS1 | 15q15 | 15 |
| Talin 2 | TLN2 | 15q15-q21 | 15 |
| SH3-domain GRB2-like 3 | SH3GL3 | 15q24 | 15 |
| Ribonuclease P 25kDa subunit | RPP25 | 15q24.1 | 15 |
| Basonuclin 1 | BNC1 | 15q25.2 | 15 |
| V-maf musculoaponeurotic fibrosarcoma oncogene homolog (avian) | MAF | 16 | 16 |
| Sulfotransferase family, cytosolic, 1A, phenol-preferring, member 3 | SULT1A3 | 16p11.2 | 16 |
| Yippee-like 3 (Drosophila) | YPEL3 | 16p11.2 | 16 |
| Mitogen-activated protein kinase 3 | MAPK3 | 16p12-p11.2 | 16 |
| Docking protein 4 | DOK4 | 16q13 | 16 |
| Gene trap locus 3 (mouse) | GTL3 | 16q21 | 16 |
| Ras-related associated with diabetes | RRAD | 16q22 | 16 |
| Cyclin-dependent kinase (CDC2-like) 10 | CDK10 | 16q24 | 16 |
| Cysteine-rich secretory protein LCCL domain containing 2 | CRISPLD2 | 16q24.1 | 16 |
| CD300 antigen like family member E | CD300LE | 17 | 17 |
| Microfibrillar-associated protein 4 | MFAP4 | 17p11.2 | 17 |
| Serpin peptidase inhibitor, clade F (alpha-2 antiplasmin, pigment epithelium derived factor), member 1 | SERPINF1 | 17p13.1 | 17 |
| Hypothetical protein MGC14376 | MGC14376 | 17p13.3 | 17 |
| Rho GTPase activating protein 23 | ARHGAP23 | 17q12 | 17 |
| Keratin 17 | KRT17 | 17q12-q21 | 17 |
| Hypothetical protein FLJ21019 | PLEKHH3 | 17q21.31 | 17 |
| Homeo box B4 | HOXB4 | 17q21-q22 | 17 |
| Myosin, light polypeptide 4, alkali; atrial, embryonic | MYL4 | 17q21-qter | 17 |
| CDC42 effector protein (Rho GTPase binding) 4 | CDC42EP4 | 17q24-q25 | 17 |
| Lethal giant larvae homolog 2 (Drosophila) | LLGL2 | 17q24-q25 | 17 |
| Solute carrier family 16 (monocarboxylic acid transporters), member 3 | SLC16A3 | 17q25 | 17 |
| G protein-coupled receptor, family C, group 5, member C | GPRC5C | 17q25 | 17 |
| Lectin, galactoside-binding, soluble, 3 binding protein | LGALS3BP | 17q25 | 17 |
| Solute carrier family 9 (sodium/hydrogen exchanger), member 3 regulator 1 | SLC9A3R1 | 17q25.1 | 17 |
| RAB37, member RAS oncogene family | RAB37 | 17q25.1 | 17 |
| Cytoglobin | CYGB | 17q25.3 | 17 |
| N-sulfoglucosamine sulfohydrolase (sulfamidase) | SGSH | 17q25.3 | 17 |
| Collectin sub-family member 12 | COLEC12 | 18pter-p11.3 | 18 |
| GATA binding protein 6 | GATA6 | 18q11.1-q11.2 | 18 |
| Collagen and calcium binding EGF domains 1 | CCBE1 | 18q21.32 | 18 |
| PQ loop repeat containing 1 | PQLC1 | 18q23 | 18 |
| Thioredoxin-like 4A | TXNL4A | 18q23 | 18 |
| Solute carrier family 27 (fatty acid transporter), member 1 | SLC27A1 | 19p13.11 | 19 |
| ATPase type 13A1 | ATP13A1 | 19p13.11 | 19 |
| MADS box transcription enhancer factor 2, polypeptide B (myocyte enhancer factor 2B) | MEF2B | 19p13.11 | 19 |
| Bone marrow stromal cell antigen 2 | BST2 | 19p13.2 | 19 |
| Jun D proto-oncogene | JUND | 19p13.2 | 19 |
| Yip1 domain family, member 2 | YIPF2 | 19p13.2 | 19 |
| Growth arrest and DNA-damage-inducible, beta | GADD45B | 19p13.3 | 19 |
| Chromosome 19 open reading frame 20 | C19orf20 | 19p13.3 | 19 |
| Chromatin modifying protein 2A | CHMP2A | 19q | 19 |
| Sirtuin (silent mating type information regulation 2 homolog) 2 (S. cerevisiae) | SIRT2 | 19q13 | 19 |
| Zinc finger protein 36, C3H type, homolog (mouse) | ZFP36 | 19q13.1 | 19 |
| SERTA domain containing 1 | SERTAD1 | 19q13.1-q13.2 | 19 |
| SERTA domain containing 3 | SERTAD3 | 19q13.2 | 19 |
| Epithelial membrane protein 3 | EMP3 | 19q13.3 | 19 |
| Myeloid-associated differentiation marker | MYADM | 19q13.41 | 19 |
| Serine palmitoyltransferase, long chain base subunit 2-like (aminotransferase 2) | SPTLC2L | 20 | 20 |
| Carboxypeptidase X (M14 family) | FAM113A | 20p13 | 20 |
| Proteasome (prosome, macropain) inhibitor subunit 1 (PI31) | PSMF1 | 20p13 | 20 |
| Mannosidase, beta A, lysosomal-like | MANBAL | 20q11.23-q12 | 20 |
| Syndecan 4 (amphiglycan, ryudocan) | SDC4 | 20q12 | 20 |
| Phosphatidylinositol glycan, class T | PIGT | 20q12-q13.12 | 20 |
| Hormonally upregulated Neu-associated kinase | HUNK | 21q22.1 | 21 |
| SH3-domain binding protein 1 | SH3BP1 | 22cen-q12.3 | 22 |
| Cysteine-rich with EGF-like domains 2 | CRELD2 | 22p13 | 22 |
| Stromal cell-derived factor 2-like 1 | SDF2L1 | 22q11.21 | 22 |
| Chromosome 22 open reading frame 5 | C22orf5 | 22q12 | 22 |
| TBC1 domain family, member 10A | TBC1D10A | 22q12.1-qter | 22 |
| Proline rich 5 (renal) | PRR5 | 22q13 | 22 |
| CDC42 effector protein (Rho GTPase binding) 1 | CDC42EP1 | 22q13.1 | 22 |
| H1 histone family, member 0 | H1F0 | 22q13.1 | 22 |
| Fibulin 1 | FBLN1 | 22q13.31 | 22 |
| Plexin B2 | PLXNB2 | 22q13.33 | 22 |
| Proteolipid protein 2 (colonic epithelium-enriched) | PLP2 | Xp11.23 | X |
| Chloride channel 5 (nephrolithiasis 2, X-linked, Dent disease) | CLCN5 | Xp11.23-p11.22 | X |
| RAB9A, member RAS oncogene family | RAB9A | Xp22.2 | X |
| Phosphorylase kinase, alpha 2 (liver) | PHKA2 | Xp22.2-p22.1 | X |
| Tafazzin (cardiomyopathy, dilated 3A (X-linked); endocardial fibroelastosis 2; Barth syndrome) | TAZ | Xq28 | X |
|  |  |  |  |
